# Vivaray Hb pro non-invasive hemoglobin device: A prospective diagnostic accuracy study measuring agreement with a calibrated blood cell counter

**DOI:** 10.1371/journal.pone.0353640

**Published:** 2026-11-03

**Authors:** Devesh Kumar, Sunita Kapoor, Ambanna Gowda, Dipti Gupta, Harsh Mittal, Suneet Sood

**Affiliations:** 1 IR Innovate Research Pvt. Ltd., Noida, Uttar Pradesh, India; 2 City X-Ray and Scan Clinic Pvt. Ltd., New Delhi, India; 3 Citizen Hospital, Kalasipalya, Bengaluru, India; 4 Dr. Dipti’s Multispeciality Clinics, Sahibabad, Uttar Pradesh, India; 5 Panchsheel Hospital, New Delhi, India; 6 Department of Surgery, IMU University, Malaysia; Menzies School of Health Research, AUSTRALIA

## Abstract

**Background:**

Non-invasive hemoglobin measurement offers a painless and rapid alternative to conventional blood-based testing. The Vivaray Hb pro is a handheld photoplethysmography-based device designed for point-of-care hemoglobin assessment without blood sampling. We evaluated the clinical performance of the Vivaray Hb pro by comparing device-generated hemoglobin values with those obtained from a calibrated laboratory blood cell counter.

**Methods:**

In this cross-sectional, non-randomized clinical performance study, participants aged ≥8 years were prospectively recruited. Hemoglobin was measured non-invasively using the Vivaray Hb pro and compared with venous blood samples analyzed on a calibrated Coulter counter. Agreement between methods was assessed using Bland–Altman analysis, including regression-based evaluation for proportional bias. Mean difference, mean absolute error (MAE) and proportions of measurements within tolerance limits were also calculated. Complete paired measurements were available for 763 individuals.

**Results:**

Bland–Altman analysis returned a mean difference of −0.40 g/dL. The bias was hemoglobin-dependent, being higher than the true value at low hemoglobin levels and underestimation at higher levels. Regression-based analysis showed proportional bias (β_1_ = −0.178), indicating decreasing difference with increasing hemoglobin concentration. The MAE was 1.5 g/dL but was lower (1.2) in the clinically predominant ranges of 8.1–13 g/dL.

**Conclusion:**

The Vivaray Hb pro can function as a useful non-invsive screening and point-of-care assessment device for measuring hemoglobin, particularly in settings where rapid, painless and repeatable measurements are desirable.

## Introduction

Anemia remains a major health problem, affecting about 30% of women of reproductive age, 35% of pregnant women, and 40% of children aged 6–59 months worldwide [1]. Prevalence is higher in low‑ and middle‑income countries, where nutritional deficiencies and infections are more common.

Screening is recommended for populations at higher risk of anemia, including pregnant women, young children, women of reproductive age, and individuals with chronic illnesses or nutritional deficiencies [2]. Early detection in pregnancy helps prevent adverse maternal and child outcomes and supports timely intervention. Global health agencies emphasize routine screening in regions with high anemia prevalence, particularly in resource‑limited settings.

A non‑invasive, point‑of‑care hemoglobin device could greatly simplify anemia screening by eliminating the need for finger‑prick or venous blood sampling. Invasive methods cause discomfort and have small but measurable risks [3], and non-invasive methods are likely to be more acceptable among children and pregnant women. They also facilitate rapid, on‑site testing in community clinics, and enable continuous monitoring where needed [4].

### Non‑invasive hemoglobin devices

Non‑invasive hemoglobin‑measuring devices have evolved substantially over the past two decades, driven by the need for rapid, painless, and point‑of‑care assessment of anemia in both clinical and community settings.

While accuracy varies across devices and populations, the overall trend shows steady improvement, supporting the role of non‑invasive technologies in anemia screening especially in children, chronic disease management, trauma care and perioperative monitoring [5,6].

The principles of non-invasive hemoglobin estimation are an extension of those of pulse oximetry. Light passed through the finger is absorbed depending on the constituents of blood, especially hemoglobin. During systole, the vessels in the finger fill up, there is more hemoglobin to absorb the light. This situation is reversed during diastole. In this way the pulse oximeter is able to detect the heart rate. Per the Beer-Lambert Law, the concentration of a given solute in a solvent can be determined by the amount of light that is absorbed by the solute at a specific wavelength [7].

Oxyhemoglobin absorbs near-infrared light at 940 nm and at 577 nm. Reduced hemoglobin absorbs light at 660 nm. It is possible to place sensors that will determine the concentration of oxygenated and reduced, and thus total hemoglobin in the blood [8,9]. The pulsatile arterial signals are filtered from non-pulsatile components (such as bone) to calculate the concentrations of the different spectrophotometric hemoglobins, and thus the device can estimate the total concentration of all hemoglobin species combined.

There are two main types of device. One uses spectrophotometry and the other uses photoplethysmography. Both use light absorption, but different sensors and algorithms. They have a bias (difference from the autoanalyzer, the gold standard) of about 0.1 to 1.1 g/dL. Spectrophotometry devices are slightly more accurate, but the photoplethysmography devices are cheaper, smaller and lighter [10–14]. While non-invasive testing does not replace laboratory blood tests, it is highly valued for its ability to show real-time trends, helping clinicians catch sudden drops in Hb faster than intermittent blood draws. Accuracy may decrease during states of low perfusion, or with persons who have very dark skin tones [15–18].

The Vivaray Hb Pro device is a photoplethysmography instrument. It is manufactured by Bosch Global Software Technologies Pvt Ltd, Bengaluru (India) (Fig 1). It is a quantitative, non-invasive device for screening and on-the-spot classification of hemoglobin levels among children (greater than 8 years) and adults in outpatient clinics, primary health setups, healthcare camps and blood transfusion centers. It is not intended for use at home.

**Fig 1:**
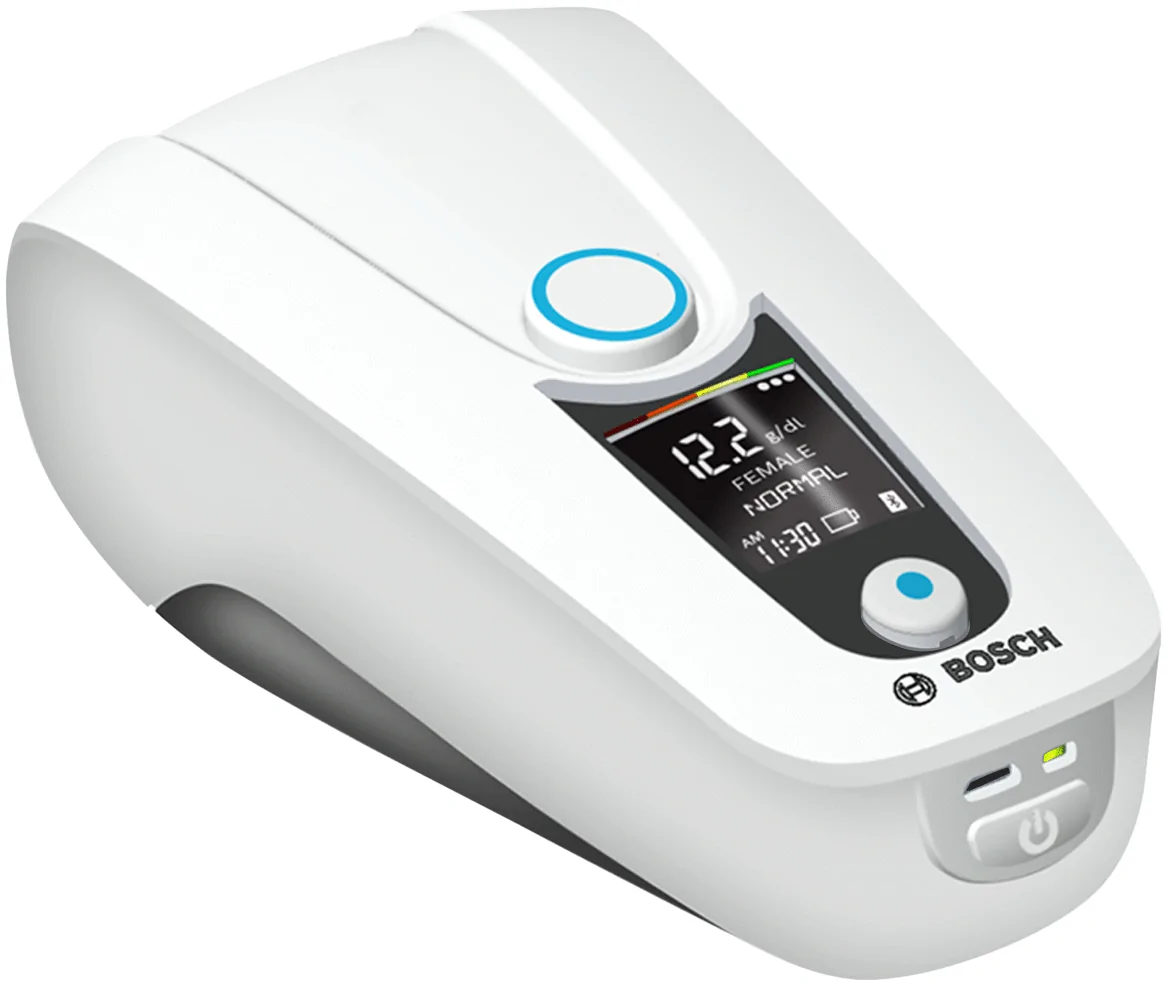
Vivaray Hb pro.

The result from Vivaray Hb pro is both quantitative and qualitative. The quantitative values for hemoglobin are shown in grams per deciliter. The qualitative results are displayed as normal, mild anemia, moderate anemia, and severe anemia (Table 1).

**Table 1:** Definitions of mild, moderate and severe anemia.

Severity of anemia	Hemoglobin level (g/dL)		
	Female	Male	Child (>8 years)
Severe	< 8	< 8	< 8
Moderate	8–10.9	8–10.9	8–10.9
Mild	11-11.9	11–12.9	11-11.9
Normal	>=12	>=13	>=12

## Objectives

The purpose of this study is to validate the clinical performance of Vivaray Hb pro and use the device generated hemoglobin value for validation by comparing it with the hemoglobin value generated from the gold standard (calibrated blood cell counter). Specifically, we intended to measure the agreement between values obtained by using this non-invasive device with venous samples measured on a calibrated blood cell counter.

## Methods

Subjects 8 years and older were prospectively recruited from the outpatient departments of the City X-ray and Scan Clinic, Dr Dipti’s Multispeciality Clinics, and Panchsheel Hospital, New Delhi, and from Citizen Hospital, Bangalore. The convenience-based recruitment started on 13-Jun-2025_and concluded on 13-Aug-2025. All the participants provided written informed consent prior to participation in the study. The informed consent process was conducted by the principal investigator, and signed consent forms were obtained before performing any study-specific activities. A copy of the signed informed consent form was provided to each participant and the original was retained in the respective participant file. For participants who were minor, written informed consent was obtained from their parents before any study-related procedures were conducted. Assent was also obtained from participants who were minor. Copies of both signed consents were provided to the participant and parent and the originals were retained in the respective participant file.

The Vivaray Hb pro device works on the principle of ‘opto-electromechanical spectrometry’. The device utilizes specific wavelengths of light to detect photoplethysmograms (PPGs) on a human finger without pricking the skin. All centers had the calibrated cell counters working on photometry (transmittance) principles. The device is designed to fit fingers 8–23 mm in thickness. The inclusion criteria were finger size 8–23 mm, age above 8 years, and hemoglobin between 4 and 18 g/dL in the sample obtained by venipuncture. A single reading was obtained per participant during the study. Fig 2 shows the flow of participants.

**Fig 2:**
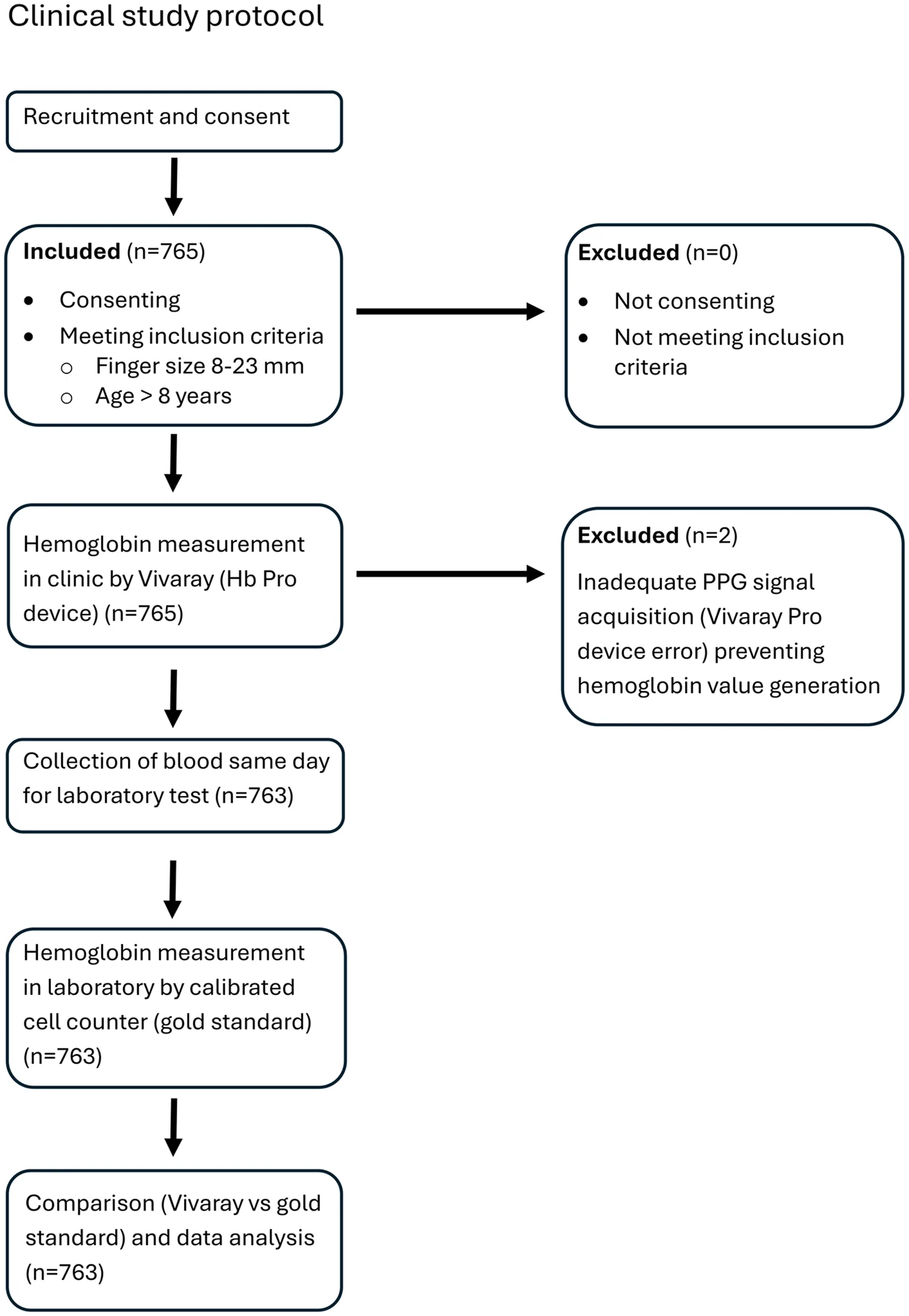
Flow chart showing subject movement and sample collection.

We excluded patients with dermatological diseases of the fingers, as well as those whose fingers were stained with nail polish or henna.

The index finger was preferred for non-invasive measurement. If the index finger was not suitable, another finger was used. Participants were instructed to place the index finger inside the finger slot, ensuring that the fingertip touched the stopper. The scan button was pressed for two seconds to begin the scanning process, which took 30–40 seconds for the measurement to be complete. At the same time 2–3 ml of blood were drawn and sent to the pathology laboratory for estimation of hemoglobin levels on a calibrated cell counter. All four laboratories were accredited by the National Accreditation Board for Testing and Calibration Laboratories (NABL), India. The names of the cell counters used for laboratory analyses were Beckman Coulter DxC700AU, Coralyzer Excel, Mindray BC-6800plus Analyzer, and Automated Hematology Analyzer Sysmex XP-300. In the laboratory the blood samples were processed by laboratory staff, not connected with the individuals who made the non-invasive readings. The corresponding hemoglobin result from the laboratory was received on the same day.

### Statistics

The sample size was calculated for a Bland-Altman analysis. We used Medcalc software to estimate sample size with the following presumptions based on our pilot tests: alpha = 0.05, beta = 0.2, expected mean of differences −0.25, expected standard deviation of differences 1.42, and maximum allowed difference between methods = 3.23. This provided a sample size of 763 subjects. Once the data was available, the reference (Coulter) was compared with the test machine (Vivaray). Agreement was estimated by the Bland-Altman method [19,20]. Calculations were carried out using R. Subjects with missing values were excluded.

### Ethics approval

The conduct of this study adhered to the rigorous ethical and regulatory standards. Ethics approvals were obtained from the following Institutional Ethics Committees: Good Society of Ethical Research (Citi X-Ray and Scan Clinic, 29 June 2024 and Dr. Dipti’s Multispeciality Clinics, 29 June 2024), Ethics Committee Panchsheel Hospital (Panchsheel Hospital, 23 July 2024), and Citizen Hospital Institutional Ethics Committee (Citizen Hospital, 29 June 2024). The study was registered with Clinical Trials Registry, India (CTRI) having the registration number: CTRI/2024/07/071199 dated 24-Jul-2024

### Reporting

This report follows the STARD guidelines (Standards for the Reporting of Diagnostic Accuracy studies) [21].

## Results

The study commenced with the recruitment of the first participant 13-Jun-2025. The last participant was measured on 13-Aug-2025. In all, 765 participants were recruited. The non-invasive hemoglobin values and the laboratory values were recorded in all cases except two, leaving 763 participants for complete analysis. The mean age was 38 years (range 17–82 years, and 81% of participants were female (Table 2)

**Table 2:** Demographic details of the participants.

Parameter	(N = 763)
**Age (Years)**	37.99 (s.d. 13.15)
**Gender**	Female 620 (81.3%), Male 143 (18.7%)
**Body Mass Index (BMI) (kg/m**^**2**^)	25.64 ± 4.24

### Agreement

Agreement was measured by a Bland-Altman analysis. The overall mean difference (test minus Coulter) was −0.40 g/dL. The mean absolute error was 1.53 g/dL (95% confidence limits 1.438, 1.626).

The mean difference varied at different hemoglobin levels. The device gave higher than true values for hemoglobin at low levels and lower than true levels in subjects with normal or high hemogolobin levels. Fig 3 plots the true hemoglobin levels against the difference for individual readings. At a true hemoglobin of 0 g/dL, the predicted bias was **8.76 g/dL** (95% CI **8.02–9.50**). The slope was **–0.77** (95% CI **–0.83 to –0.71**), meaning that the bias declined by roughly **0.77 g/dL** for every 1 g/dL increase in true hemoglobin.

**Fig 3:**
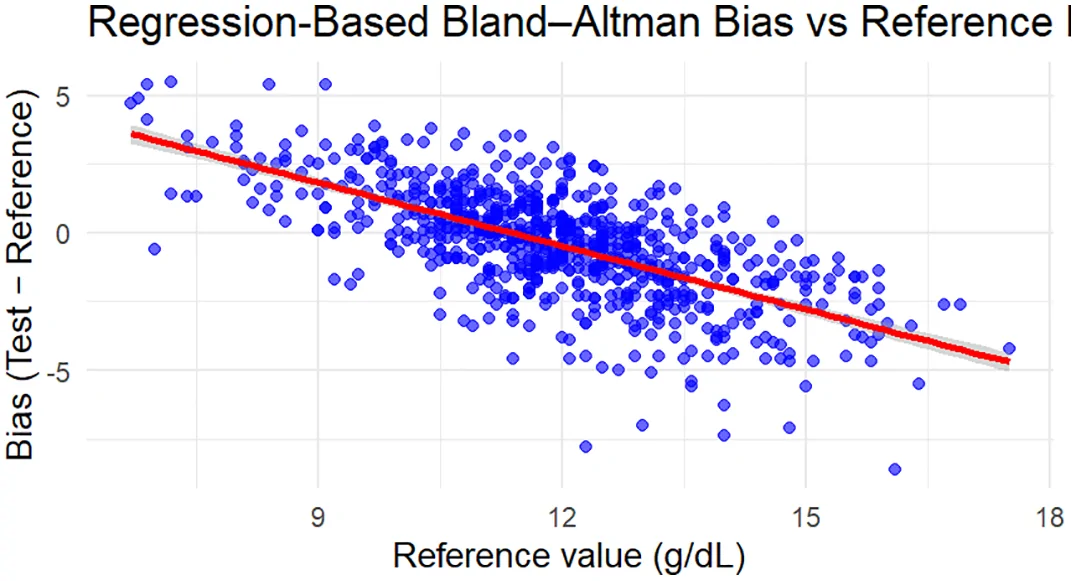
Bias at different hemoglobin levels. The shaded area around the regression line represents the 95% confidence interval of the estimated difference.

Since the mean difference varied at different hemoglobin levels, we carried out a regression-based Bland-Altman analysis (Fig 4) The intercept (β_0_) was 1.68, and the regression slope (β_1_) was −0.178. The regression‑based Bland–Altman analysis showed proportional bias. Its slope (β_1_) was −0.178 (95% confidence limits −0.29, −0.07), meaning that for every for every 1 g/dL increase in the mean hemoglobin, the difference (reference minus test) changed by −0.178 g/dL. The clinically relevant bias varies with the measurement level according to the equation “bias(x) = b0 + b1x”. The test overestimated hemoglobin concentrations at low hemoglobin levels, and underestimated the concentrations at high levels. We also calculated the values separately for females and males. For females the value for intercept and slope were 2.11 and −0.22. For males the corresponding values were 5.94 and −0.47.

**Fig 4:**
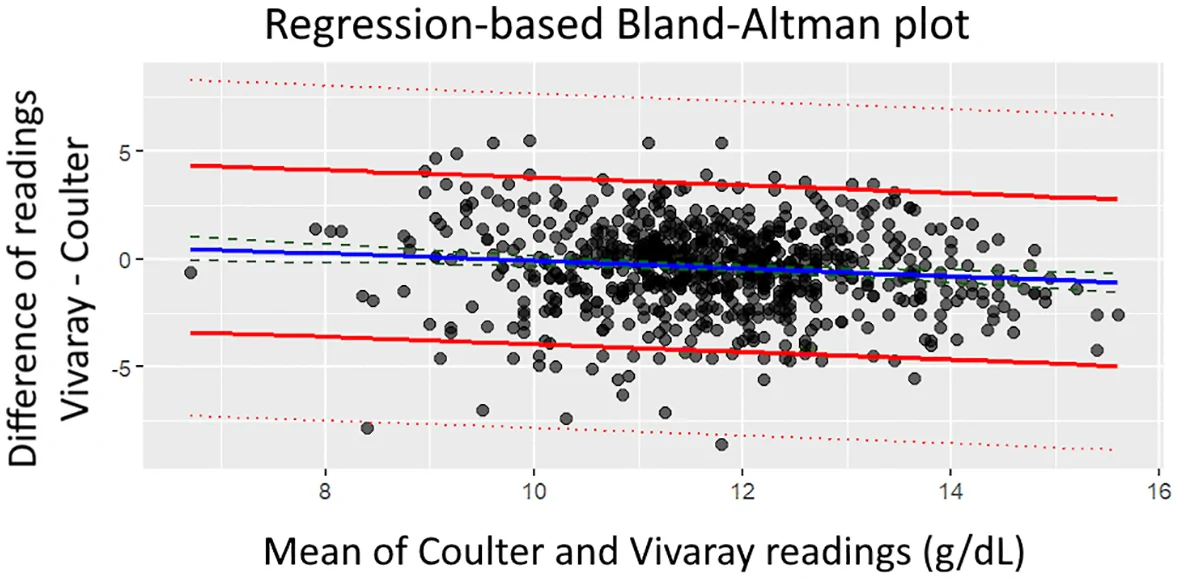
Regression-based Bland–Altman Plot: Bosch Device vs Coulter Reference. In this figure, the solid blue line represents the fitted regression line, and the adjacent dashed lines represent the 95% confidence limits around that regression line (the uncertainty around the regression‑estimated bias). The solid red lines represent the regression-based limits of agreement, and the dotted red lines represent the 95% confidence limits for the limits of agreement. The regression intercept is 1.68 (95% confidence limits 2.96, 0.40). The intercept is numerically equal to the predicted difference at mean = 0 but can also be interpreted as the adjusted mean difference after accounting for proportional bias.

The regression-based predicted bias at hemoglobin 8, 11, 12 and 13 (cutoff levels for severity of anemia) was 0.26, −0.28, −0.45 and −0.63 respectively (Table 3).

**Table 3:** Predicted regression-based bias at different hemoglobin values.

Hemoglobin level	Predicted bias	Limits of Agreement
8	0.26 (−0.17, 0.68)	−3.61, 4.12
11	−0.28 (−0.44,-0.12)	−4.14, 3.59
12	−0.45 (−0.60, −0.31)	−4.32, 3.41
13	−0.63 (−0.83, −0.43)	−4.49, 3.23

The figures in parentheses are the 95% confidence limits for the bias.

We also calculated the bias at different levels of severity (Table 4). The bias was 3.15 for subjects categorized as severe anemia, but was much lower in the other groups (Table 4).

Finally, we assessed heteroscedasticity by regressing the absolute measurement error on the mean hemoglobin value. The heteroscedasticity coefficient (slope) was −0.071 (95% CI −0.143 to 0.002; p = 0.057), indicating that the variability of the measurement error did not change significantly across the hemoglobin range.

**Table 4:** Regression-based bias at different levels of severity of anemia.

Severity of anemia	Predicted bias	Limits of agreement
Severe anemia (n=12)	3.15 (2.79, 3.53)	1.93, 4.36
Moderate anemia (n=186)	1.1 (0.91, 1.29)	−1.45, 3.65
Mild anemia (n=224)	−0.02 (−0.11, 0.08)	−1.39, 1.35
Normal (n=341)	−1.6 (−1.77, −1.43)	−4.77, 1.57

This table gives the mean difference at different degrees of anemia. The categories severe, moderate, mild and normal are described in Table 1. The figures in parentheses are the confidence limits for the bias.

## Discussion

It has been recognized since the early 1970s that oxygenated and deoxygenated hemoglobin absorb light differently at specific wavelengths [22]. This principle underlies modern pulse oximetry, in which a photodetector measures light at two wavelengths (660 nm and 940 nm). By separating the pulsatile arterial component from the non‑pulsatile tissue signal using Aoyagi’s ratio‑of‑ratios method, the device estimates arterial oxygen saturation.

Non‑invasive hemoglobin measurement extends this concept by using multiple wavelengths to spectrally resolve different hemoglobin species, producing a continuous estimate of total hemoglobin through pulse co‑oximetry. The first clinically useful devices employing this technology were introduced by Masimo in 2008–2009 [23]. Hemoglobin concentrations determined by this spectrophotometric method are usually termed SpHb to distinguish them from the total hemoglobin concentration obtained in a laboratory from venipuncture samples.

Non-invasive hemoglobin monitoring using pulse co-oximetry has demonstrated clinical utility across a broad range of care settings. It has been tested in an outpatient setting [17,24], emergency department [25], in neonates [26] and in intensive care units [4,15]. Noninvasive measurement significantly reduces the anxiety associated with venipuncture [25], and seems likely to provide the benefit of continuous measurement [4]. The devices have been used often in operation theaters [27–32], where there seems to be a trend towards improved decision-making regarding blood transfusions [15,27,28,31].

This large, prospective, cross-sectional clinical performance study demonstrates that the Vivaray Hb pro is capable of providing non-invasive hemoglobin estimates with acceptable agreement when compared with a calibrated laboratory blood cell counter. The device showed an overall mean absolute error of 1.53 g/dL, but a mean absolute error of only 1.25 g/dL in the clinically predominant range of 8.1 to 13 g/dL. The overall mean difference was −0.4 g/dL. Most measurements fall within clinically relevant tolerance limits.

Since only single readings were taken from the subjects, there is no precision or repeatability data at present.

The performance of this device is similar to that of other noninvasive instruments. Noninvasive devices have been used in different settings. Moreira et al [33] reviewed their use in the operation theater. Of 39 reviewed papers the bias varied between −1.27 and +2.28 g/dL, being positive in half and negative in half of all reports. Among the reports of positive bias the mean bias was 0.598 g/dL. Among the papers showing negative bias, the mean value was −0.564 g/dL. In the outpatient setting, where the Vivaray device was also used, other devices also give similar results. Young and coworkers [34] used three different devices for initial health screening of pediatric and adult refugees in Atlanta, Georgia. The bias values with the HemoCue® and the Masimo Pronto® devices were 0.4 g/dL (limits of agreement ± 1.4) and −0.4 g/dL (± 3.0) respectively. Smarphone applications gave slightly poorer agreement, being −1.6 (± 4) for the Apple® and −0.7 (± 4) for the Android® instruments.

The bias varies at different hemoglobin levels, as seen in Fig 2 and Table 3 above. This observed pattern of hemoglobin dependent bias – characterized by overestimation at lower hemoglobin levels and underestimation at higher levels – is commonly seen with non-invasive devices, a phenomenon termed by Denisenko et al [35] as a “regression to the mean”. Several workers have observed this effect [15,33,35–37]. However, this phenomenon also means that at the clinically relevant mid-range of the hemoglobin concentration spectrum the bias is much smaller, as is evident from Table 4.

To see if the precision of the machine had improved over the years we looked at the correlation between year of publication and width of the 95% confidence limits of the Bland-Altman limits of agreement in the studies (Fig 5).

**Fig 5:**
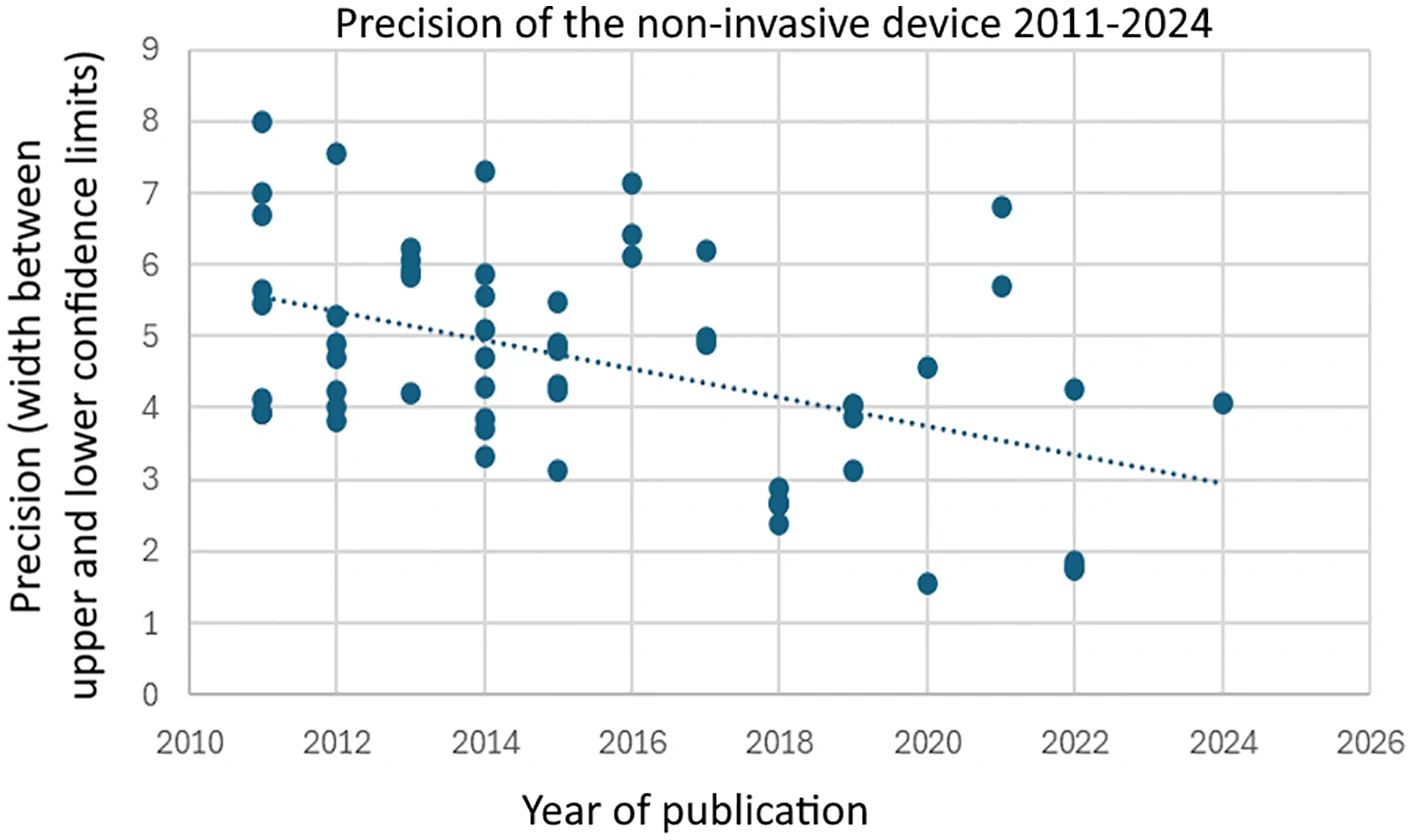
Precision of non-invasive measurement over the years. This figure is an analysis of the tables in a recent systematic review. [33].

Pearson’s coefficient of correlation was −0.46 (95% confidence limits −0.64, −0.24), indicating that since 2011 there has been a small but statistically significant improvement in the precision of the devices.

Given that the bias is low at the clinically relevant hemoglobin ranges, the literature is generally supportive of the use of pulsed co-oximetry in both acute care [27,28,31,33], as well as in the outpatient setting [17,36,38–40]. That said, some reports have urged caution in the use of noninvasive devices at present, given the extent of the bias [25,29,30,41,42]. The concerns are that the wide limits of agreement cast doubt on the clinical usefulness of such devices. If transfusion is being considered, other testing methods may be required.

## Conclusions

The results support the use of the Vivaray Hb pro as a non-invasive hemoglobin screening and point-of-care assessment tool, particularly in settings where rapid, painless, and repeatable measurements are desirable. While laboratory hemoglobin estimation remains essential for definitive diagnosis and clinical decision-making, the Vivaray Hb pro offers a practical solution for initial screening, on the spot classification, and in outpatient clinics, primary healthcare facilities, and community screening programs. The device therefore represents a valuable adjunct to conventional testing methods in efforts to improve anemia screening and early identification in resource constrained and high-throughput environments.

### Strengths and weaknesses

A key strength of this investigation is the large sample size of 763 participants, making it one of the larger real-world evaluations of a non-invasive hemoglobin device conducted in outpatient and community healthcare settings. The use of Bland–Altman analysis, complemented by a regression-based approach to account for proportional bias, provides a robust and transparent assessment of agreement across the hemoglobin range. The prospective data collection, simultaneous non-invasive and venous measurements, and inclusion of participants across a wide range of hemoglobin values further strengthen the reliability and generalizability of the findings.

While this study is one of its kind to have a greater sample size compared to the studies of other non-invasive hemoglobin devices published studies, the authors would like to highlight the areas of improvement for future studies. Firstly, although individuals aged eight years and older were eligible for inclusion, the study was not designed or powered to permit a dedicated subgroup analysis for pediatric populations; therefore, conclusions regarding device performance in children remain limited. Secondly, repeated measurements were not performed, precluding evaluation of within subject variability or test–retest reliability. Future studies incorporating broader demographic representation, targeted pediatric cohorts, repeated measurements, and other real-world deployment scenarios would further strengthen the evidence base and help refine the clinical role of non-invasive hemoglobin screening technologies.

## Supporting information

S1 File Supporting information for Tables and Figures. (DOCX)

## References

[1] World Health Organization. WHO Global Anaemia Estimates, 2025 Edition. https://www.who.int/data/gho/data/themes/topics/anaemia_in_women_and_children. 2025. Accessed 2026 February 20.

[2] WHO global anaemia estimates: key findings. Geneva: World Health Organization. 2025. https://iris.who.int/server/api/core/bitstreams/ffbc07c6-420c-4711-8589-2a7b6f3a3da2/content

[3] Sutikno T, Handayani L, Ruliyandari R, Wijaya O, Satrian PH, Arsadiando W. Review of recent advances in non-invasive hemoglobin estimation. IAES Int J Artif Intell. 2025;14(2):1031.

[4] Frasca D, Dahyot-Fizelier C, Catherine K, Levrat Q, Debaene B, Mimoz O. Accuracy of a continuous noninvasive hemoglobin monitor in intensive care unit patients. Crit Care Med. 2011;39(10):2277–82. doi: 10.1097/CCM.0b013e3182227e2d 21666449

[5] Joseph B, Haider A, Rhee P. Non-invasive hemoglobin monitoring. Int J Surg. 2016;33(Pt B):254–7. doi: 10.1016/j.ijsu.2015.11.048 26654895

[6] Tang B, Yu X, Xu L, Zhu A, Zhang Y, Huang Y. Continuous noninvasive hemoglobin monitoring estimates timing for detecting anemia better than clinicians: a randomized controlled trial. BMC Anesthesiol. 2019;19(1):80. doi: 10.1186/s12871-019-0755-1 31101083 PMC6525351

[7] Kyriacou P, Budidha K, Abay TY. Optical Techniques for Blood and Tissue Oxygenation. Encyclopedia of Biomedical Engineering. Elsevier. 2019. p. 461–72. doi: 10.1016/b978-0-12-801238-3.10886-4

[8] Chan ED, Chan MM, Chan MM. Pulse oximetry: understanding its basic principles facilitates appreciation of its limitations. Respir Med. 2013;107(6):789–99. doi: 10.1016/j.rmed.2013.02.004 23490227

[9] Jubran A. Pulse oximetry. Crit Care. 2015;19(1):272. doi: 10.1186/s13054-015-0984-8 26179876 PMC4504215

[10] Okazaki K, Okazaki K, Uesugi M, Matsusima T, Hataya H. Evaluation of the accuracy of a non-invasive hemoglobin-monitoring device in schoolchildren. Pediatr Neonatol. 2022;63(1):19–24. doi: 10.1016/j.pedneo.2021.05.028 34389262

[11] Peng F, Zhang N, Chen C, Wu F, Wang W. Ensemble Extreme Learning Machine Method for Hemoglobin Estimation Based on PhotoPlethysmoGraphic Signals. Sensors. 2024;24(6):1736. doi: 10.3390/s24061736 38543999 PMC10974404

[12] R R, S P, A S KD, J B J. Non-invasive hemoglobin measurement using optical method. Heliyon. 2024;10(15):e35777. doi: 10.1016/j.heliyon.2024.e35777 39170470 PMC11337058

[13] Song Y. Non-invasive hemoglobin level estimation using photoplethysmography with performance comparison of advanced ML algorithms. SIViP. 2025;19(6). doi: 10.1007/s11760-025-04040-8

[14] Zhu J, Sun R, Liu H, Wang T, Cai L, Chen Z. A non-invasive hemoglobin detection device based on multispectral photoplethysmography. Biosensors. 2023;14(1):0. doi: 10.3390/bios14010022 38248399 PMC11154563

[15] Helmer P, Steinisch A, Hottenrott S, Schlesinger T, Sammeth M, Meybohm P, et al. Evaluation of Non-Invasive Hemoglobin Monitoring in Perioperative Patients: A Retrospective Study of the Rad-67TM (Masimo). Diagnostics (Basel). 2025;15(2):128. doi: 10.3390/diagnostics15020128 39857014 PMC11763668

[16] Johansson A, Öberg PÅ. Estimation of haemoglobin concentration in human whole blood with spectrophotometry. Med Biol Eng Comput. 1999;37(3). doi: 10.1007/BF02513328

[17] Shah N, Osea EA, Martinez GJ. Accuracy of noninvasive hemoglobin and invasive point-of-care hemoglobin testing compared with a laboratory analyzer. Int J Lab Hematol. 2014;36(1):56–61. doi: 10.1111/ijlh.12118 23809685 PMC4232003

[18] Yassen K, Omer N, Alsahaf F, Al Amer F, Alhamad F, Alherz I, et al. J Clin Med. 2023;12(24):7517. doi: 10.3390/jcm12247517 38137588 PMC10744205

[19] Bland JM, Altman DG. Statistical methods for assessing agreement between two methods of clinical measurement. Lancet. 1986;1(8476):307–10. 2868172

[20] Bland JM, Altman DG. Measuring agreement in method comparison studies. Stat Methods Med Res. 1999;8(2):135–60. doi: 10.1177/096228029900800204 10501650

[21] Bossuyt PM, Reitsma JB, Bruns DE, Gatsonis CA, Glasziou PP, Irwig L, et al. STARD 2015: an updated list of essential items for reporting diagnostic accuracy studies. BMJ. 2015;351:h5527. doi: 10.1136/bmj.h5527 26511519 PMC4623764

[22] Severinghaus JW. Takuo Aoyagi: discovery of pulse oximetry. Anesth Analg. 2007;105(6 Suppl):S1–4. doi: 10.1213/01.ane.0000269514.31660.09 18048890

[23] Miller RD, Ward TA, Shiboski SC, Cohen NH. A comparison of three methods of hemoglobin monitoring in patients undergoing spine surgery. Anesth Analg. 2011;112(4):858–63. doi: 10.1213/ANE.0b013e31820eecd1 21385985

[24] Raikhel M. Accuracy of noninvasive and invasive point-of-care total blood hemoglobin measurement in an outpatient setting. Postgrad Med. 2012;124(4):250–5. doi: 10.3810/pgm.2012.07.2584 22913913

[25] Gayat E, Aulagnier J, Matthieu E, Boisson M, Fischler M. Non-invasive measurement of hemoglobin: assessment of two different point-of-care technologies. PLoS One. 2012;7(1):e30065. doi: 10.1371/journal.pone.0030065 22238693 PMC3253126

[26] Panda SK, Mishra A, Jena PK. Agreement between Noninvasive Hemoglobin and Laboratory Hemoglobin Measurements in Neonates: A Systematic Review and Meta-Analysis. Neonatology. 2023;120(1):24–32. doi: 10.1159/000526100 36450265

[27] Awada WN, Mohmoued MF, Radwan TM, Hussien GZ, Elkady HW. Continuous and noninvasive hemoglobin monitoring reduces red blood cell transfusion during neurosurgery: A prospective cohort study. J Clin Monit Comput. 2015;29(6):733–40. doi: 10.1007/s10877-015-9660-4 25649717 PMC4621711

[28] Berkow L, Rotolo S, Mirski E. Continuous noninvasive hemoglobin monitoring during complex spine surgery. Anesth Analg. 2011;113(6):1396–402. doi: 10.1213/ANE.0b013e318230b425 21965372

[29] Butwick A, Hilton G, Carvalho B. Non-invasive haemoglobin measurement in patients undergoing elective Caesarean section. Br J Anaesth. 2012;108(2):271–7. doi: 10.1093/bja/aer373 22116296

[30] Dewhirst E, Naguib A, Winch P, Rice J, Galantowicz M, McConnell P, et al. Accuracy of noninvasive and continuous hemoglobin measurement by pulse co-oximetry during preoperative phlebotomy. J Intensive Care Med. 2014;29(4):238–42. doi: 10.1177/0885066613485355 23753242

[31] Ehrenfeld JM, Henneman JP. Continuous Non-invasive Hemoglobin Monitoring during Orthopedic Surgery: A Randomized Trial. J Blood Disord Transfus. 2014;05(09). doi: 10.4172/2155-9864.1000237

[32] Lamhaut L, Apriotesei R, Combes X, Lejay M, Carli P, Vivien B. Comparison of the accuracy of noninvasive hemoglobin monitoring by spectrophotometry (SpHb) and HemoCue® with automated laboratory hemoglobin measurement. Anesthesiology. 2011;115(3):548–54. doi: 10.1097/ALN.0b013e3182270c22 21716091

[33] Moreira L, Engelman E, Estruch-Pons I, Parvais M, Lecucq A, Martens B, et al. Non-invasive vs biological blood determination of haemoglobinemia for perioperative management: A systematic review with meta-analysis. J Clin Monit Comput. 2025;39(6):1113–35. doi: 10.1007/s10877-025-01323-w 40739388

[34] Young MF, Raines K, Jameel F, Sidi M, Oliveira-Streiff S, Nwajei P, et al. Non-invasive hemoglobin measurement devices require refinement to match diagnostic performance with their high level of usability and acceptability. PLoS One. 2021;16(7):e0254629. doi: 10.1371/journal.pone.0254629 34270591 PMC8284642

[35] Denisenko G, Khvan O, Bardadin I, Shirshin E, Yakimov B. Non-invasive hemoglobin assessment via RGB-imaging: The impact of physiology and interpatient variability on the method accuracy. J Innov Opt Health Sci. 2026;19(05). doi: 10.1142/s1793545825400048

[36] Alwabari M, Alhamad F, Alsahaf F, Al Amer F, Alniniya F, Alherz I, et al. Can non-invasive spectrophotometric hemoglobin replace laboratory hemoglobin concentrations for preoperative anemia screening? A diagnostic test accuracy study. J Clin Med. 2023;12(17):5733. doi: 10.3390/jcm12175733 37685800 PMC10488634

[37] Koech A, Mwaniki I, Mutunga J, Mukhanya M, Mwadime E, Ochieng M, et al. Diagnostic accuracy of a non-invasive spot-check hemoglobin meter, Masimo Rad-67® pulse CO-Oximeter®, in detection of anemia in antenatal care settings in Kenya. Front Glob Womens Health. 2024;5:1427261. doi: 10.3389/fgwh.2024.1427261 39469077 PMC11513392

[38] Amano I, Murakami A. Use of non-invasive total hemoglobin measurement as a screening tool for anemia in children. Pediatr Int. 2013;55(6):803–5. doi: 10.1111/ped.12236 24330295

[39] Dangwilailert R, Lekmak S, Promlee D, Pongkunakorn T, Permpikul P. Evaluation of non-invasive hemoglobin measurement in blood donors at a tertiary care hospital, Bangkok, Thailand. PLoS One. 2026;21(5):e0348447. doi: 10.1371/journal.pone.0348447 42096441 PMC13152161

[40] Das Mahapatra PP, Roy C, Agarwal K, Banerjee J, Sharma S. Performance of the non-invasive point-of-care device, EzeCheck, for haemoglobin assessment in adults and children in community and institutional care settings. PLOS Digit Health. 2024;3(5):e0000500. doi: 10.1371/journal.pdig.0000500 38717987 PMC11078411

[41] Ahankari AS, Fogarty AW, Tata LJ, Dixit JV, Myles PR. Assessment of a non-invasive haemoglobin sensor NBM 200 among pregnant women in rural India. BMJ Innov. 2016;2(2):70–7. doi: 10.1136/bmjinnov-2015-000085

[42] Kim S-H, Lilot M, Murphy LS-L, Sidhu KS, Yu Z, Rinehart J, et al. Accuracy of continuous noninvasive hemoglobin monitoring: A systematic review and meta-analysis. Anesth Analg. 2014;119(2):332–46. doi: 10.1213/ANE.0000000000000272 24914627

