# Supplementary material for: Vivaray Hb pro non-invasive hemoglobin device: A prospective diagnostic accuracy study measuring agreement with a calibrated blood cell counter
Source: PLoS One. 2026 Nov 3;21(11):e0353640. doi: 10.1371/journal.pone.0353640 (PMC13730371; doi:10.1371/journal.pone.0353640)
Supplement: Supplementary file 1 [file pone.0353640.s001.docx]

**Supporting information**

**Table 1. Definitions of mild, moderate and severe anemia**

**Table 2. Demographic details of the participants**

**Table 3. Predicted bias at different hemoglobin values**

**Table 4: Regression-based bias at different levels of severity of anemia.**

This table gives the mean difference at different degrees of anemia. The categories severe, moderate, mild and normal are described in Table 1. The figures in parentheses are the confidence limits for the bias.

**Fig 1. Vivaray Hb pro**

**Fig 2. Flow chart showing subject movement and sample collection.**

**Fig 3. Bias at different hemoglobin levels.**

The shaded area around the regression line represents the 95% confidence interval of the estimated difference.

**Fig 4. Regression-based Bland–Altman Plot: Bosch Device vs Coulter Reference**

In this figure, the solid blue line represents the fitted regression line, and the adjacent dashed lines represent the 95% confidence limits around that regression line (the uncertainty around the regression‑estimated bias). The solid red lines represent the regression-based limits of agreement, and the dotted red lines represent the 95% confidence limits for the limits of agreement. The regression intercept is 1.68 (95% confidence limits 2.96, 0.40). The intercept is numerically equal to the predicted difference at mean = 0 but can also be interpreted as the adjusted mean difference after accounting for proportional bias.

**Fig 5. Precision of non-invasive measurement over the years.**

This figure is an analysis of the tables in a recent systematic review. [33]
