# Supplementary material for: Vivaray Hb pro non-invasive hemoglobin device: A prospective diagnostic accuracy study measuring agreement with a calibrated blood cell counter
Source: PLoS One. 2026 Nov 3;21(11):e0353640. doi: 10.1371/journal.pone.0353640 (PMC13730371; doi:10.1371/journal.pone.0353640)
Supplement: Supplementary file 2 [file pone.0353640.s002.docx]

## Review of the manuscript: ‘Vivaray Hb pro noninvasive hemoglobin device: a prospective diagnostic accuracy study measuring agreement with a calibrated blood cell counter’ (PONE-D-26-25852)

This article assesses the agreement between a noninvasive hemoglobin measurement device and the gold standard laboratory CBC in measuring hemoglobin levels. The study was methodologically sound and provided an excellent report of the findings, complemented by a comprehensive introduction that gives readers sufficient background to understand the study's context. However, the discussion section (outside the Conclusion and Strengths and Weaknesses subheadings) seems to stray from the study's findings and needs a rework.

### Major Comments

- The discussion should mainly focus on the findings of this study and put them into context around the broader non-invasive Hb monitoring landscape.
- Some of the questions to consider for discussion: Is it “better” than the other non-invasive assays? Is this systematic bias also observed in other assays? Will these findings be reported to the manufacturer? Is this device fit for use? What use is it fit for? What, if any, is the clinical relevance of the observed systematic bias?
- The only discussion about the findings was in the last paragraph.
- I would suggest integrating the first paragraph of the Conclusions as the beginning of the Discussion section, and adding discussions about relevant literature to each point mentioned in this paragraph.
- The discussion section should guide the readers to understand why, even with the systematic bias, the assessed device was still concluded to be suitable for certain kinds of utility by the authors. As it currently stands, the authors leave readers to draw their own conclusions by providing separate descriptions of the findings and the realities of other non-invasive Hb devices on the market.

### Comments

- Abstract
  - The comment or interpretation section of the abstract should at least explain what the findings mean, in the context of Hb measurement.
- Introduction
  - Second to last paragraph “The Vivaray Hb Pro device is a photoplethysmography instrument.”: Kindly add the manufacturer name and country in a bracket after the device’s name.
- Methods
  - Having a photograph or a diagram of the device would be most helpful for the readers
  - Figure 1: consider including details of exclusion reasons (age 8 years and below, etc.) to the figure
  - “At the same time blood was drawn and sent to the pathology laboratory …”: venous blood? How much blood was taken?
  - “… calibrated cell counter”: please include the details of the instruments used in this study. Coulter was referenced in the sections below this one, but it should be clearly stated.
- References
  - Reference 1: Can you be so kind as to cite the report published by the WHO (reference 2) instead of the web page?
