# Supplementary material for: Vivaray Hb pro non-invasive hemoglobin device: A prospective diagnostic accuracy study measuring agreement with a calibrated blood cell counter
Source: PLoS One. 2026 Nov 3;21(11):e0353640. doi: 10.1371/journal.pone.0353640 (PMC13730371; doi:10.1371/journal.pone.0353640)
Supplement: Supplementary file 3 [file pone.0353640.s003.docx]

**Summary:**

This article presents a prospective clinical performance evaluation of the Vivaray Hb Pro, a non-invasive photoplethysmography-based hemoglobin device, comparing its measurements with laboratory hemoglobin values in 763 individuals. The study addresses a critical clinical need for point‑of‑care anemia screening, is adequately powered, and uses appropriate agreement analysis, including Bland–Altman plots with regression to assess proportional bias.

Overall, the article is clinically valuable and well written. However, several key issues require clarification before it can be considered for publication. Kindly see below some points for consideration:

**Major comments:**

- The objective of the study was to validate the clinical performance of the Vivaray Hb Pro; however, the analytical approach used reflects a method‑comparison analysis between two measurement techniques. Please provide precision, sensitivity, specificity, and repeatability results to support the clinical validation of the Vivaray Hb device.
- The study was registered with the Clinical Trials Registry–India (CTRI) under registration number CTRI/2024/07/071199 dated 24‑Jul‑2024. According to the registry, the age inclusion criteria were 11–65 years; however, the article reports data collection from individuals aged 8 years or older. Please clarify this discrepancy.
- Figure 1: Please include the corresponding numbers in the flowchart (e.g., number screened, number excluded, number included, and the number not meeting each criterion).
- The flowchart indicates that inclusion and exclusion criteria were assessed after obtaining consent, yet participants were excluded for failing to consent. This is contradictory and needs revision for clarity.
- In the Results section, only proportional bias is reported. It is important to also present additional regression diagnostics, including residual patterns, heteroscedasticity assessment, and other relevant parameters.
- The manuscript states that mean differences varied across hemoglobin levels and suggests that the device may help identify mild, moderate, and severe anemia. It is important to stratify the analysis accordingly to demonstrate performance across these anemia categories.
- When comparing a device with a gold‑standard method, reporting diagnostic accuracy metrics—sensitivity, specificity, PPV, NPV, and AUC—is essential.
- As most participants were female and adults, subgroup analyses by sex and age group are recommended.
- Please provide the 95% confidence intervals for the MAE, intercept, and slope (Figure 2), along with the corresponding p‑values.
- The Methods and Statistics sections should be revised to reflect these analytical requirements and ensure methodological transparency.

**Minor comments:**

- In the abstract, replace “comment” with “conclusion.”
- Please replace “I” with “in” in the section Non‑invasive hemoglobin devices.
- Please provide the manufacturer’s name and details of the calibrated blood cell counter.
- Report g/dL consistently throughout the manuscript.
- Please provide a clearer explanation or definition of the terms “overestimation at low Hb” and “underestimation at high Hb”, including their methodological and clinical implications.
- Please clarify whether the Vivaray Hb Pro operator and the gold‑standard test operator were the same individual. Also specify the time interval between the two measurements.
- Figure order: Figure 3 appears before Figure 2; please revise to follow chronological sequence.
- Figure 2: Replace the placeholder variable x with the actual variable name (e.g., hemoglobin measured with the Vivaray Hb Pro).
- Correct the sentence in the Methods section from “participants who was minor” to “participants who were minors.”
- Remove the repeated word “for for” in the Methods section.
- Use “non‑invasive” consistently throughout the manuscript, including in the title.
- Provide the explanation to consider upper values as 0, 9 and 15. I would suggest matching with the Hb classification.
