# Supplementary material for: Vivaray Hb pro non-invasive hemoglobin device: A prospective diagnostic accuracy study measuring agreement with a calibrated blood cell counter
Source: PLoS One. 2026 Nov 3;21(11):e0353640. doi: 10.1371/journal.pone.0353640 (PMC13730371; doi:10.1371/journal.pone.0353640)
Supplement: Supplementary file 4 [file pone.0353640.s004.docx]

23 Aug 2026

Response to reviewers

Thank you for the detailed and careful review of the paper. The text in red font in this document refers to the recommendations not completely followed. We have made the changes for nearly all recommendations. A notable exception is the suggestion for adding sensitivity and specificity data, for which we have not made the changes in the manuscript. The reasons are given under Reviewer 2 Major comments.

Suneet Sood (corresponding author)

I. **From the editorial office**
1. Thank you for stating the following in the Competing Interests section:

“I have read the journal's policy and the authors of this manuscript have the following competing interests:

Devesh Kumar and Suneet Sood are consultants for Think-i, Unit of Tenet Health Edutech Pvt. Ltd., the Clinical Research Organization that conducted the study. ”

We note that one or more of the authors are employed by a commercial company: “Think-i, Unit of Tenet Health Edutech Pvt. Ltd.”

1. Please provide an amended Funding Statement declaring this commercial affiliation, as well as a statement regarding the Role of Funders in your study. If the funding organization did not play a role in the study design, data collection and analysis, decision to publish, or preparation of the manuscript and only provided financial support in the form of authors' salaries and/or research materials, please review your statements relating to the author contributions, and ensure you have specifically and accurately indicated the role(s) that these authors had in your study. You can update author roles in the Author Contributions section of the online submission form.

Please also include the following statement within your amended Funding Statement.

“The funder provided support in the form of salaries for authors “D.K., S.S.” but did not have any additional role in the study design, data collection and analysis, decision to publish, or preparation of the manuscript. The specific roles of these authors are articulated in the ‘author contributions’ section.”

If your commercial affiliation did play a role in your study, please state and explain this role within your updated Funding Statement.

**Answer**: *We have made the required changes. The statement “The funder provided …author contributions’ section.” has been added to the Conflicts section of the manuscript (now renamed “Competing interests”). In the Editorial Manager, the sections relating to funding have been edited.*

2. Please also provide an updated Competing Interests Statement declaring this commercial affiliation along with any other relevant declarations relating to employment, consultancy, patents, products in development, or marketed products, etc.

Within your Competing Interests Statement, please confirm that this commercial affiliation does not alter your adherence to all PLOS ONE policies on sharing data and materials by including the following statement: "This does not alter our adherence to  PLOS ONE policies on sharing data and materials.” (as detailed online in our guide for authors http://journals.plos.org/plosone/s/competing-interests) . If this adherence statement is not accurate and  there are restrictions on sharing of data and/or materials, please state these. Please note that we cannot proceed with consideration of your article until this information has been declared.

**Answer**: *The sentence “This does not alter our adherence to  PLOS ONE policies on sharing data and materials.” has been added to the Conflicts section of the manuscript (now renamed “Competing interests”). This sentence has also been added to our answer in the section Funded Studies in the Editorial manager.*

II. **Review #1 Comments to the Author**
1. The study was methodologically sound and provided an excellent report of the findings, complemented by a comprehensive introduction that gives readers sufficient background to understand the study's context. However, the discussion section (outside the Conclusion and Strengths and Weaknesses subheadings) seems to stray from the study's findings and needs a rework.

***Answer****: We have looked at the Discussion section. We would be happy to edit it but are unable to determine exactly the areas that need rework.*

III. **Reviewer #2:**

Summary: This article presents a prospective clinical performance evaluation of the Vivaray Hb Pro, a non-invasive photoplethysmography-based hemoglobin device, comparing its measurements with laboratory hemoglobin values in 763 individuals. The study addresses a critical clinical need for point of care anemia screening, is adequately powered, and uses appropriate agreement analysis, including Bland–Altman plots with regression to assess proportional bias.
Overall, the article is clinically valuable and well written. However, several key issues require clarification before it can be considered for publication. Kindly see below some points for consideration:

**Reviewer #2 Major comments:**

2. The objective of the study was to validate the clinical performance of the Vivaray Hb Pro; however, the analytical approach used reflects a method‑comparison analysis between two measurement techniques. Please provide precision, sensitivity, specificity, and repeatability results to support the clinical validation of the Vivaray Hb device.

***Answer****: With regards to sensitivity, specificity and ROC, we did indeed calculate the values and write them in our first draft. Here is the data:*

| **Group** | **TP** | **FP** | **FN** | **TN** | **Total** | **Sensitivity** | **Specificity** |
| --- | --- | --- | --- | --- | --- | --- | --- |
| Females | 272 | 164 | 87 | 97 | 620 | 0.76 | 0.37 |
| Males | 28 | 32 | 35 | 48 | 143 | 0.44 | 0.6 |
| All | 300 | 196 | 122 | 145 | 763 | 0.71 | 0.43 |

*We also have the data for different levels of anemia, as defined in Table 1 in the manuscript.*

ROC: All cases

*The ROC curve was also generated separately for females as well (AUC 0.402).*

*However, sensitivity and specificity data has been removed from the final manuscript. We respectfully submit that while sensitivity and specificity are good markers for analyzing dichotomous variables, they are not suitable for diagnostic machines with outputs that are continuous variables. If we impose a clinical decision threshold, the result indeed becomes binary (anemia vs not anemia), and sensitivity/ specificity become meaningful. However, setting a decision threshold makes the results very misleading. If, for example, the reference standard returns a value of 11.9 g/dl for a female, and the test device returns a value of 12.1, the test device would be very accurate, but its output would be a false negative. The appropriate accuracy metrics for a device of this sort are the Bland–Altman limits of agreement. We urge the reviewers to accept our contention that sensitivity, specificity and AUC metrics be excluded from the paper.*

*The reviewers are, however, quite correct in asking for repeatability and precision data. However, this data was not captured in the study, and cannot be provided. The final sentence of the Discussion section reflects this weakness.*

3. The study was registered with the Clinical Trials Registry–India (CTRI) under registration number CTRI/2024/07/071199 dated 24‑Jul‑2024. According to the registry, the age inclusion criteria were 11–65 years; however, the article reports data collection from individuals aged 8 years or older. Please clarify this discrepancy.

**Answer:** *The registry document also says that the age criterion was above the age of 8 years. The registry document has been uploaded in the Editorial Manager, filename “REGISTRY - CTRI_29-Jul-2024_Bosch Study.pdf”*

4. Figure 1: Please include the corresponding numbers in the flowchart (e.g., number screened, number excluded, number included, and the number not meeting each criterion).

***Answer:*** *The flow chart has been updated. A revised version of the flow chart, filename Figure 1 flow chart version 2.tiff, has also been uploaded to the Editorial Manager.*

5. The flowchart indicates that inclusion and exclusion criteria were assessed after obtaining consent, yet participants were excluded for failing to consent. This is contradictory and needs revision for clarity.

**Answer**: *Thank you for pointing this out. In fact, two participants were screened but not included in the study because adequate photoplethysmography (PPG) signals could not be acquired by the Vivaray Hb Pro device. The device displayed a "Signal Error" message, and a hemoglobin value could not be generated for these participants. The flow chart has been modified. No participants declined consent.*

6. In the Results section, only proportional bias is reported. It is important to also present additional regression diagnostics, including residual patterns, heteroscedasticity assessment, and other relevant parameters.

***Answer****: We performed only one test per subject, and do not have precision or repeatability data. A comment to that effect has been made in the final sentence of the Discussion. We have commented on the residuals and heteroscedasticity in the Results, after Table 4, in the final paragraph of the Results section.*

7. The manuscript states that mean differences varied across hemoglobin levels and suggests that the device may help identify mild, moderate, and severe anemia. It is important to stratify the analysis accordingly to demonstrate performance across these anemia categories.

***Answer****: We have added a table that states the bias and the limits of agreement for severe, moderate, mild anemia and normal levels. This is table 4 in the results.*

8. When comparing a device with a gold‑standard method, reporting diagnostic accuracy metrics—sensitivity, specificity, PPV, NPV, and AUC—is essential.

***Answer****: Please refer to the comment above about sensitivity, specificity and AUC (item no 2).*

9. As most participants were female and adults, subgroup analyses by sex and age group are recommended.

***Answer****: We have added information about the values for males and females in the Results, in the text after Figure 2.*

10. Please provide the 95% confidence intervals for the MAE, intercept, and slope (Figure 2), along with the corresponding p‑values. The Methods and Statistics sections should be revised to reflect these analytical requirements and ensure methodological transparency.

***Answer****: The MAE was 1.53 g/dL (95% confidence limits 1.438, 1.626). this information has been provided in the “Agreement” paragraph”. For the regression-based analysis, the intercept was 1.68 (95% confidence limits 2.96, 0.40). This information is provided in the legend to Figure 3. The slope for the regression-based Bland-Altman analysis (β₁) was -0.178 (95% confidence limits -0.29, -0.07). This information is provided in the paragraph that follows Figure 2.*

**Reviewer #2 Minor comments:**

11. In the abstract, replace “comment” with “conclusion.” **Answer**: *Done*
12. Please replace “I” with “in” in the section Non‑invasive hemoglobin devices. **Answer**: *Done*
13. Please provide the manufacturer’s name and details of the calibrated blood cell counter. **Answer**: *The information has been provided in paragraph 4 of the Methods, immediately prior to the section on Statistics.*
14. Report g/dL consistently throughout the manuscript. **Answer**: *Done*
15. Please provide a clearer explanation or definition of the terms “overestimation at low Hb” and “underestimation at high Hb”, including their methodological and clinical implications. **Answer**: *A small edit has been made in the abstract and in the Results*
16. Please clarify whether the Vivaray Hb Pro operator and the gold‑standard test operator were the same individual. Also specify the time interval between the two measurements.

**Answer**: *The Vivaray Hb Pro operator and the gold‑standard test operator were different individuals. It takes approximately 30-40 secs for the measurement to complete in Vivaray Hb Pro device. The blood sample for the gold standard test was processed separately, and the corresponding hemoglobin result from the laboratory was received on the same day. This information has been entered into the Methods section in paragraph 4 of the Methods, immediately prior to the “Statistics” section.*

17. Figure order: Figure 3 appears before Figure 2; please revise to follow chronological sequence. **Answer**: *There was a typographical error. This has been corrected.*
18. Figure 2: Replace the placeholder variable x with the actual variable name (e.g., hemoglobin measured with the Vivaray Hb Pro). **Answer**: We could not identify the error. Figure 2 seems okay. However, we have uploaded a newer version of Figure 2.
19. Correct the sentence in the Methods section from “participants who was minor” to “participants who were minors.” **Answer**: *Done.*
20. Remove the repeated word “for for” in the Methods section. **Answer:** *Done*
21. Use “non‑invasive” consistently throughout the manuscript, including in the title. **Answer:** *Edited*
22. Provide the explanation to consider upper values as 0, 9 and 15. I would suggest matching with the Hb classification. **Answer**: *Agreed. Table 3 has been modified accordingly.*
