# Supplementary material for: Vivaray Hb pro non-invasive hemoglobin device: A prospective diagnostic accuracy study measuring agreement with a calibrated blood cell counter
Source: PLoS One. 2026 Nov 3;21(11):e0353640. doi: 10.1371/journal.pone.0353640 (PMC13730371; doi:10.1371/journal.pone.0353640)
Supplement: Supplementary file 6 [file pone.0353640.s006.docx]

5 October 2026

Editor

PLOS ONE

Sub: Paper for publication – Fifth revision

This refers to our paper titled "Vivaray Hb pro noninvasive hemoglobin device: a prospective diagnostic accuracy study measuring agreement with a calibrated blood cell counter". (We had earlier neglected to see the comments made by Reviewer 1, and had only responded to Reviewer 2.)

Here are our point-by-point answers to the issues raised by Reviewer 1:

| Review of the manuscript: ‘Vivaray Hb pro noninvasive hemoglobin device: a prospective diagnostic accuracy study measuring agreement with a calibrated blood cell counter’ (PONE-D-26-25852) | |
| --- | --- |
| This article assesses the agreement between a noninvasive hemoglobin measurement device and the gold standard laboratory CBC in measuring hemoglobin levels. The study was methodologically sound and provided an excellent report of the findings, complemented by a comprehensive introduction that gives readers sufficient background to understand the study's context. However, the discussion section (outside the Conclusion and Strengths and Weaknesses subheadings) seems to stray from the study's findings and needs a rework. | |
| **Major Comments** | **Our answers** |
| •       The discussion should mainly focus on the findings of this study and put them into context around the broader non-invasive Hb monitoring landscape. | *Yes, we agree that this would improve the Discussion. We have made changes.* |
| •       Some of the questions to consider for discussion: Is it “better” than the other non-invasive assays? Is this systematic bias also observed in other assays? Will these findings be reported to the manufacturer? Is this device fit for use? What use is it fit for? What, if any, is the clinical relevance of the observed systematic bias? | *Thank you for the observations. We have modified the text to align with these suggestions. Specifically, we have answered the question  a. about the quality of the device compared to others (Paragraph beginning with the words "The performance of this device is similar to that of other noninvasive instruments:,  b. about the fitness of this device for use (Paragraph beginning with the words "Given that the bias is low at the clinically relevant..."), and c. clinical relevance of the systematic bias (Paragraph beginning with the words "The bias varies at different hemoglobin levels...")* |
| •       The only discussion about the findings was in the last paragraph. | *True! Apologies for the limited connection between findings and the discussion.* |
| •       I would suggest integrating the first paragraph of the Conclusions as the beginning of the Discussion section, and adding discussions about relevant literature to each point mentioned in this paragraph. | *Agreed. We have moved the paragraph to a location early in the discussion. The relocated paragraph still begins with the words "This large, prospective…"* |
| •       The discussion section should guide the readers to understand why, even with the systematic bias, the assessed device was still concluded to be suitable for certain kinds of utility by the authors. As it currently stands, the authors leave readers to draw their own conclusions by providing separate descriptions of the findings and the realities of other non-invasive Hb devices on the market. | *Agreed. We think that with the edits the Discussion should have become better.* |
| •       **Abstract** |  |
| o   The comment or interpretation section of the abstract should at least explain what the findings mean, in the context of Hb measurement. | *The comments section of the abstract does state the implication of the device. We have made a slight edit, to make the conclusion a little clearer. The paragraph immediately prior to the comments section provides several relevant values, as also the statement about the bias in the clinically predominant range. This should allow readers to decide whether they are comfortable with the precision of the instrument.* |
| •       **Introduction** |  |
| o   Second to last paragraph “The Vivaray Hb Pro device is a photoplethysmography instrument.”: Kindly add the manufacturer name and country in a bracket after the device’s name. | *Reviewer 2 made the same comment. We have added manufacturer information. The text in the* ***Introduction*** *now adds the following sentence: It is manufactured by Bosch Global Software Technologies Pvt Ltd, Bengaluru (India).* |
| •       Methods |  |
| o   Having a photograph or a diagram of the device would be most helpful for the readers | *The photograph has been inserted. As corresponding and senior author I confess I am ambivalent about this suggestion. I was wondering if insertion of the picture might add an advertising flavor to the paper, but after consideration the picture has been put in.* |
| o   Figure 1: consider including details of exclusion reasons (age 8 years and below, etc.) to the figure | *The figure (now Figure 2) has been modified accordingly* |
| o   “At the same time blood was drawn and sent to the pathology laboratory …”: venous blood? How much blood was taken? | *The Methods section now says "At the same time 2 to 3 ml of blood was drawn and sent to the pathology laboratory" instead of "At the same time blood was drawn and sent to the pathology laboratory". This line is written in the paragraph immediately prior to the Statistics section.* |
| o   “… calibrated cell counter”: please include the details of the instruments used in this study. Coulter was referenced in the sections below this one, but it should be clearly stated. | *Reviewer 2 had made the same observation. We have added information about the instruments.* |
| •       References |  |
| o   Reference 1: Can you be so kind as to cite the report published by the WHO (reference 2) instead of the web page? | *One citation (WHO) was copied directly from the paper, from the paragraph titled "Suggested citation".*  *The other WHO paper has been edited.*  *The two WHO references now read as follows:*  1. WHO Global Anaemia estimates, 2025 Edition [Internet]. World Health Organization; 2025.; 2025 [cited 2026 Feb 20]. Available from: https://www.who.int/data/gho/data/themes/topics/anaemia_in_women_and_children  2. WHO global anaemia estimates: key findings, 2025. [Internet]. Geneva: World Health Organization; 2025.; 2025. Available from: https://iris.who.int/server/api/core/bitstreams/ffbc07c6-420c-4711-8589-2a7b6f3a3da2/content |
