# Supplementary material for: Vivaray Hb pro non-invasive hemoglobin device: A prospective diagnostic accuracy study measuring agreement with a calibrated blood cell counter
Source: PLoS One. 2026 Nov 3;21(11):e0353640. doi: 10.1371/journal.pone.0353640 (PMC13730371; doi:10.1371/journal.pone.0353640)
Supplement: Peer review history

# Peer review: Decision Letter 0

**DOI:** 10.1371/journal.pone.0353640.r001

**Authors:** Benedikt Ley

18 Jul 2026

Dear Dr. Sood,

plosone@plos.org. When you're ready to submit your revision, log on to https://www.editorialmanager.com/pone/ and select the 'Submissions Needing Revision' folder to locate your manuscript file.

- A letter that responds to each point raised by the academic editor and reviewer(s). You should upload this letter as a separate file labeled 'Response to Reviewers'.
- A marked-up copy of your manuscript that highlights changes made to the original version. You should upload this as a separate file labeled 'Revised Manuscript with Track Changes'.
- An unmarked version of your revised paper without tracked changes. You should upload this as a separate file labeled 'Manuscript'.

If you would like to make changes to your financial disclosure, please include your updated statement in your cover letter. Guidelines for resubmitting your figure files are available below the reviewer comments at the end of this letter.

If applicable, we recommend that you deposit your laboratory protocols in protocols.io to enhance the reproducibility of your results. Protocols.io assigns your protocol its own identifier (DOI) so that it can be cited independently in the future. For instructions see: https://journals.plos.org/plosone/s/submission-guidelines#loc-laboratory-protocols. Additionally, PLOS ONE offers an option for publishing peer-reviewed Lab Protocol articles, which describe protocols hosted on protocols.io. Read more information on sharing protocols at https://plos.org/protocols?utm_medium=editorial-email&utm_source=authorletters&utm_campaign=protocols.

As the corresponding author, your ORCID iD is verified in the submission system and will appear in the published article. PLOS supports the use of ORCID, and we encourage all coauthors to register for an ORCID iD and use it as well. Please encourage your coauthors to verify their ORCID iD within the submission system before final acceptance, as unverified ORCID iDs will not appear in the published article. *Only*  the individual author can complete the verification step; PLOS staff *cannot*  verify ORCID iDs on behalf of authors.

We look forward to receiving your revised manuscript.

Kind regards,

Benedikt Ley, PhD

Academic Editor

PLOS One

Journal Requirements:

When submitting your revision, we need you to address these additional requirements.

1.Please ensure that your manuscript meets PLOS ONE's style requirements, including those for file naming. The PLOS ONE style templates can be found at

https://journals.plos.org/plosone/s/file?id=wjVg/PLOSOne_formatting_sample_main_body.pdf and

https://journals.plos.org/plosone/s/file?id=ba62/PLOSOne_formatting_sample_title_authors_affiliations.pdf

2. Please note that PLOS One has specific guidelines on code sharing for submissions in which author-generated code underpins the findings in the manuscript. In these cases, we expect all author-generated code to be made available without restrictions upon publication of the work. Please review our guidelines at https://journals.plos.org/plosone/s/materials-and-software-sharing#loc-sharing-code and ensure that your code is shared in a way that follows best practice and facilitates reproducibility and reuse.

3. We note that the grant information you provided in the ‘Funding Information’ and ‘Financial Disclosure’ sections do not match.

When you resubmit, please ensure that you provide the correct grant numbers for the awards you received for your study in the ‘Funding Information’ section.

4. Thank you for stating the following in the Competing Interests section:

“I have read the journal's policy and the authors of this manuscript have the following competing interests:

Devesh Kumar and Suneet Sood are consultants for Think-i, Unit of Tenet Health Edutech Pvt. Ltd., the Clinical Research Organization that conducted the study. ”

We note that one or more of the authors are employed by a commercial company: “Think-i, Unit of Tenet Health Edutech Pvt. Ltd.”

1. Please provide an amended Funding Statement declaring this commercial affiliation, as well as a statement regarding the Role of Funders in your study. If the funding organization did not play a role in the study design, data collection and analysis, decision to publish, or preparation of the manuscript and only provided financial support in the form of authors' salaries and/or research materials, please review your statements relating to the author contributions, and ensure you have specifically and accurately indicated the role(s) that these authors had in your study. You can update author roles in the Author Contributions section of the online submission form.

Please also include the following statement within your amended Funding Statement.

“The funder provided support in the form of salaries for authors “D.K., S.S.” but did not have any additional role in the study design, data collection and analysis, decision to publish, or preparation of the manuscript. The specific roles of these authors are articulated in the ‘author contributions’ section.”

If your commercial affiliation did play a role in your study, please state and explain this role within your updated Funding Statement.

2. Please also provide an updated Competing Interests Statement declaring this commercial affiliation along with any other relevant declarations relating to employment, consultancy, patents, products in development, or marketed products, etc.

Within your Competing Interests Statement, please confirm that this commercial affiliation does not alter your adherence to all PLOS ONE policies on sharing data and materials by including the following statement: "This does not alter our adherence to  PLOS ONE policies on sharing data and materials.” (as detailed online in our guide for authors http://journals.plos.org/plosone/s/competing-interests) . If this adherence statement is not accurate and  there are restrictions on sharing of data and/or materials, please state these. Please note that we cannot proceed with consideration of your article until this information has been declared.

Please include both an updated Funding Statement and Competing Interests Statement in your cover letter. We will change the online submission form on your behalf.

5. Thank you for uploading your study's underlying data set. Unfortunately, the repository you have noted in your Data Availability statement does not qualify as an acceptable data repository according to PLOS's standards.

At this time, please upload the minimal data set necessary to replicate your study's findings to a stable, public repository (such as figshare or Dryad) and provide us with the relevant URLs, DOIs, or accession numbers that may be used to access these data. For a list of recommended repositories and additional information on PLOS standards for data deposition, please see https://journals.plos.org/plosone/s/recommended-repositories.

6. Please include captions for your Supporting Information files at the end of your manuscript, and update any in-text citations to match accordingly. Please see our Supporting Information guidelines for more information: http://journals.plos.org/plosone/s/supporting-information.

7. If the reviewer comments include a recommendation to cite specific previously published works, please review and evaluate these publications to determine whether they are relevant and should be cited. There is no requirement to cite these works unless the editor has indicated otherwise.

Additional Editor Comments:

Kindly adress all comments made by reviewers 1 and 2.

[Note: HTML markup is below. Please do not edit.]

Reviewer's Responses to Questions

**Comments to the Author**

1. Is the manuscript technically sound, and do the data support the conclusions?

Reviewer #1: Yes

Reviewer #2: Partly

**********

2. Has the statistical analysis been performed appropriately and rigorously?

Reviewer #1: Yes

Reviewer #2: Yes

**********

3. Have the authors made all data underlying the findings in their manuscript fully available?

The PLOS Data policy

Reviewer #1: Yes

Reviewer #2: Yes

**********

4. Is the manuscript presented in an intelligible fashion and written in standard English?

Reviewer #1: Yes

Reviewer #2: Yes

**********

Reviewer #1: The study was methodologically sound and provided an excellent report of the findings, complemented by a comprehensive introduction that gives readers sufficient background to understand the study's context. However, the discussion section (outside the Conclusion and Strengths and Weaknesses subheadings) seems to stray from the study's findings and needs a rework.

Reviewer #2: Summary:

This article presents a prospective clinical performance evaluation of the Vivaray Hb Pro, a non-invasive photoplethysmography-based hemoglobin device, comparing its measurements with laboratory hemoglobin values in 763 individuals. The study addresses a critical clinical need for point of care anemia screening, is adequately powered, and uses appropriate agreement analysis, including Bland–Altman plots with regression to assess proportional bias.

Overall, the article is clinically valuable and well written. However, several key issues require clarification before it can be considered for publication. Kindly see below some points for consideration:

Major comments:

• The objective of the study was to validate the clinical performance of the Vivaray Hb Pro; however, the analytical approach used reflects a method‑comparison analysis between two measurement techniques. Please provide precision, sensitivity, specificity, and repeatability results to support the clinical validation of the Vivaray Hb device.

• The study was registered with the Clinical Trials Registry–India (CTRI) under registration number CTRI/2024/07/071199 dated 24‑Jul‑2024. According to the registry, the age inclusion criteria were 11–65 years; however, the article reports data collection from individuals aged 8 years or older. Please clarify this discrepancy.

• Figure 1: Please include the corresponding numbers in the flowchart (e.g., number screened, number excluded, number included, and the number not meeting each criterion).

• The flowchart indicates that inclusion and exclusion criteria were assessed after obtaining consent, yet participants were excluded for failing to consent. This is contradictory and needs revision for clarity.

• In the Results section, only proportional bias is reported. It is important to also present additional regression diagnostics, including residual patterns, heteroscedasticity assessment, and other relevant parameters.

• The manuscript states that mean differences varied across hemoglobin levels and suggests that the device may help identify mild, moderate, and severe anemia. It is important to stratify the analysis accordingly to demonstrate performance across these anemia categories.

• When comparing a device with a gold‑standard method, reporting diagnostic accuracy metrics—sensitivity, specificity, PPV, NPV, and AUC—is essential.

• As most participants were female and adults, subgroup analyses by sex and age group are recommended.

• Please provide the 95% confidence intervals for the MAE, intercept, and slope (Figure 2), along with the corresponding p‑values.

• The Methods and Statistics sections should be revised to reflect these analytical requirements and ensure methodological transparency.

Minor comments:

• In the abstract, replace “comment” with “conclusion.”

• Please replace “I” with “in” in the section Non‑invasive hemoglobin devices.

• Please provide the manufacturer’s name and details of the calibrated blood cell counter.

• Report g/dL consistently throughout the manuscript.

• Please provide a clearer explanation or definition of the terms “overestimation at low Hb” and “underestimation at high Hb”, including their methodological and clinical implications.

• Please clarify whether the Vivaray Hb Pro operator and the gold‑standard test operator were the same individual. Also specify the time interval between the two measurements.

• Figure order: Figure 3 appears before Figure 2; please revise to follow chronological sequence.

• Figure 2: Replace the placeholder variable x with the actual variable name (e.g., hemoglobin measured with the Vivaray Hb Pro).

• Correct the sentence in the Methods section from “participants who was minor” to “participants who were minors.”

• Remove the repeated word “for for” in the Methods section.

• Use “non‑invasive” consistently throughout the manuscript, including in the title.

• Provide the explanation to consider upper values as 0, 9 and 15. I would suggest matching with the Hb classification.

**********

what does this mean?). If published, this will include your full peer review and any attached files.

If you choose “no”, your identity will remain anonymous but your review may still be made public.

**Do you want your identity to be public for this peer review?**  For information about this choice, including consent withdrawal, please see our Privacy Policy

Reviewer #1: No

Reviewer #2: No

**********

[NOTE: If reviewer comments were submitted as an attachment file, they will be attached to this email and accessible via the submission site. Please log into your account, locate the manuscript record, and check for the action link "View Attachments". If this link does not appear, there are no attachment files.]

To ensure your figures meet our technical requirements, please review our figure guidelines: https://journals.plos.org/plosone/s/figures

You may also use PLOS’s free figure tool, NAAS, to help you prepare publication quality figures: https://journals.plos.org/plosone/s/figures#loc-tools-for-figure-preparation.

NAAS will assess whether your figures meet our technical requirements by comparing each figure against our figure specifications.

Attachment Submitted filename: Review PONE_D_26-25852.docx

Attachment Submitted filename: Reviewer comments.docx

# Author comment: Author response to Decision Letter 1

**DOI:** 10.1371/journal.pone.0353640.r002

25 Aug 2026

(Please refer to the uploaded file titled "Response to Reviwers". The formatting, and a diagram, will not show in this box.)

I. From the editorial office

1. Thank you for stating the following in the Competing Interests section:

“I have read the journal's policy and the authors of this manuscript have the following competing interests:

Devesh Kumar and Suneet Sood are consultants for Think-i, Unit of Tenet Health Edutech Pvt. Ltd., the Clinical Research Organization that conducted the study. ”

We note that one or more of the authors are employed by a commercial company: “Think-i, Unit of Tenet Health Edutech Pvt. Ltd.”

1. Please provide an amended Funding Statement declaring this commercial affiliation, as well as a statement regarding the Role of Funders in your study. If the funding organization did not play a role in the study design, data collection and analysis, decision to publish, or preparation of the manuscript and only provided financial support in the form of authors' salaries and/or research materials, please review your statements relating to the author contributions, and ensure you have specifically and accurately indicated the role(s) that these authors had in your study. You can update author roles in the Author Contributions section of the online submission form.

Please also include the following statement within your amended Funding Statement.

“The funder provided support in the form of salaries for authors “D.K., S.S.” but did not have any additional role in the study design, data collection and analysis, decision to publish, or preparation of the manuscript. The specific roles of these authors are articulated in the ‘author contributions’ section.”

If your commercial affiliation did play a role in your study, please state and explain this role within your updated Funding Statement.

Answer: We have made the required changes. The statement “The funder provided …author contributions’ section.” has been added to the Conflicts section of the manuscript (now renamed “Competing interests”). In the Editorial Manager, the sections relating to funding have been edited.

2. Please also provide an updated Competing Interests Statement declaring this commercial affiliation along with any other relevant declarations relating to employment, consultancy, patents, products in development, or marketed products, etc.

Within your Competing Interests Statement, please confirm that this commercial affiliation does not alter your adherence to all PLOS ONE policies on sharing data and materials by including the following statement: "This does not alter our adherence to PLOS ONE policies on sharing data and materials.” (as detailed online in our guide for authors http://journals.plos.org/plosone/s/competing-interests) . If this adherence statement is not accurate and there are restrictions on sharing of data and/or materials, please state these. Please note that we cannot proceed with consideration of your article until this information has been declared.

Answer: The sentence “This does not alter our adherence to PLOS ONE policies on sharing data and materials.” has been added to the Conflicts section of the manuscript (now renamed “Competing interests”). This sentence has also been added to our answer in the section Funded Studies in the Editorial manager.

II. Review #1 Comments to the Author

1. The study was methodologically sound and provided an excellent report of the findings, complemented by a comprehensive introduction that gives readers sufficient background to understand the study's context. However, the discussion section (outside the Conclusion and Strengths and Weaknesses subheadings) seems to stray from the study's findings and needs a rework.

Answer: We have looked at the Discussion section. We would be happy to edit it but are unable to determine exactly the areas that need rework.

III. Reviewer #2:

Summary: This article presents a prospective clinical performance evaluation of the Vivaray Hb Pro, a non-invasive photoplethysmography-based hemoglobin device, comparing its measurements with laboratory hemoglobin values in 763 individuals. The study addresses a critical clinical need for point of care anemia screening, is adequately powered, and uses appropriate agreement analysis, including Bland–Altman plots with regression to assess proportional bias.

Overall, the article is clinically valuable and well written. However, several key issues require clarification before it can be considered for publication. Kindly see below some points for consideration:

Reviewer #2 Major comments:

2. The objective of the study was to validate the clinical performance of the Vivaray Hb Pro; however, the analytical approach used reflects a method comparison analysis between two measurement techniques. Please provide precision, sensitivity, specificity, and repeatability results to support the clinical validation of the Vivaray Hb device.

Answer: With regards to sensitivity, specificity and ROC, we did indeed calculate the values and write them in our first draft. Here is the data:

Group TP FP FN TN Total Sensitivity Specificity

Females 272 164 87 97 620 0.76 0.37

Males 28 32 35 48 143 0.44 0.6

All 300 196 122 145 763 0.71 0.43

We also have the data for different levels of anemia, as defined in Table 1 in the manuscript.

ROC: All cases

The ROC curve was also generated separately for females as well (AUC 0.402).

However, sensitivity and specificity data has been removed from the final manuscript. We respectfully submit that while sensitivity and specificity are good markers for analyzing dichotomous variables, they are not suitable for diagnostic machines with outputs that are continuous variables. If we impose a clinical decision threshold, the result indeed becomes binary (anemia vs not anemia), and sensitivity/ specificity become meaningful. However, setting a decision threshold makes the results very misleading. If, for example, the reference standard returns a value of 11.9 g/dl for a female, and the test device returns a value of 12.1, the test device would be very accurate, but its output would be a false negative. The appropriate accuracy metrics for a device of this sort are the Bland–Altman limits of agreement. We urge the reviewers to accept our contention that sensitivity, specificity and AUC metrics be excluded from the paper.

The reviewers are, however, quite correct in asking for repeatability and precision data. However, this data was not captured in the study, and cannot be provided. The final sentence of the Discussion section reflects this weakness.

3. The study was registered with the Clinical Trials Registry–India (CTRI) under registration number CTRI/2024/07/071199 dated 24 Jul 2024. According to the registry, the age inclusion criteria were 11–65 years; however, the article reports data collection from individuals aged 8 years or older. Please clarify this discrepancy.

Answer: The registry document also says that the age criterion was above the age of 8 years. The registry document has been uploaded in the Editorial Manager, filename “REGISTRY - CTRI_29-Jul-2024_Bosch Study.pdf”

4. Figure 1: Please include the corresponding numbers in the flowchart (e.g., number screened, number excluded, number included, and the number not meeting each criterion).

Answer: The flow chart has been updated. A revised version of the flow chart, filename Figure 1 flow chart version 2.tiff, has also been uploaded to the Editorial Manager.

5. The flowchart indicates that inclusion and exclusion criteria were assessed after obtaining consent, yet participants were excluded for failing to consent. This is contradictory and needs revision for clarity.

Answer: Thank you for pointing this out. In fact, two participants were screened but not included in the study because adequate photoplethysmography (PPG) signals could not be acquired by the Vivaray Hb Pro device. The device displayed a "Signal Error" message, and a hemoglobin value could not be generated for these participants. The flow chart has been modified. No participants declined consent.

6. In the Results section, only proportional bias is reported. It is important to also present additional regression diagnostics, including residual patterns, heteroscedasticity assessment, and other relevant parameters.

Answer: We performed only one test per subject, and do not have precision or repeatability data. A comment to that effect has been made in the final sentence of the Discussion. We have commented on the residuals and heteroscedasticity in the Results, after Table 4, in the final paragraph of the Results section.

7. The manuscript states that mean differences varied across hemoglobin levels and suggests that the device may help identify mild, moderate, and severe anemia. It is important to stratify the analysis accordingly to demonstrate performance across these anemia categories.

Answer: We have added a table that states the bias and the limits of agreement for severe, moderate, mild anemia and normal levels. This is table 4 in the results.

8. When comparing a device with a gold standard method, reporting diagnostic accuracy metrics—sensitivity, specificity, PPV, NPV, and AUC—is essential.

Answer: Please refer to the comment above about sensitivity, specificity and AUC (item no 2).

9. As most participants were female and adults, subgroup analyses by sex and age group are recommended.

Answer: We have added information about the values for males and females in the Results, in the text after Figure 2.

10. Please provide the 95% confidence intervals for the MAE, intercept, and slope (Figure 2), along with the corresponding p values. The Methods and Statistics sections should be revised to reflect these analytical requirements and ensure methodological transparency.

Answer: The MAE was 1.53 g/dL (95% confidence limits 1.438, 1.626). this information has been provided in the “Agreement” paragraph”. For the regression-based analysis, the intercept was 1.68 (95% confidence limits 2.96, 0.40). This information is provided in the legend to Figure 3. The slope for the regression-based Bland-Altman analysis (β) was -0.178 (95% confidence limits -0.29, -0.07). This information is provided in the paragraph that follows Figure 2.

Reviewer #2 Minor comments:

11. In the abstract, replace “comment” with “conclusion.” Answer: Done

12. Please replace “I” with “in” in the section Non invasive hemoglobin devices. Answer: Done

13. Please provide the manufacturer’s name and details of the calibrated blood cell counter. Answer: The information has been provided in paragraph 4 of the Methods, immediately prior to the section on Statistics.

14. Report g/dL consistently throughout the manuscript. Answer: Done

15. Please provide a clearer explanation or definition of the terms “overestimation at low Hb” and “underestimation at high Hb”, including their methodological and clinical implications. Answer: A small edit has been made in the abstract and in the Results

16. Please clarify whether the Vivaray Hb Pro operator and the gold standard test operator were the same individual. Also specify the time interval between the two measurements.

Answer: The Vivaray Hb Pro operator and the gold standard test operator were different individuals. It takes approximately 30-40 secs for the measurement to complete in Vivaray Hb Pro device. The blood sample for the gold standard test was processed separately, and the corresponding hemoglobin result from the laboratory was received on the same day. This information has been entered into the Methods section in paragraph 4 of the Methods, immediately prior to the “Statistics” section.

17. Figure order: Figure 3 appears before Figure 2; please revise to follow chronological sequence. Answer: There was a typographical error. This has been corrected.

18. Figure 2: Replace the placeholder variable x with the actual variable name (e.g., hemoglobin measured with the Vivaray Hb Pro). Answer: We could not identify the error. Figure 2 seems okay. However, we have uploaded a newer version of Figure 2.

19. Correct the sentence in the Methods section from “participants who was minor” to “participants who were minors.” Answer: Done.

20. Remove the repeated word “for for” in the Methods section. Answer: Done

21. Use “non invasive” consistently throughout the manuscript, including in the title. Answer: Edited

22. Provide the explanation to consider upper values as 0, 9 and 15. I would suggest matching with the Hb classification. Answer: Agreed. Table 3 has been modified accordingly.

Attachment Submitted filename: Response to reviewers.docx

# Peer review: Decision Letter 1

**DOI:** 10.1371/journal.pone.0353640.r003

**Authors:** Benedikt Ley

4 Sep 2026

Dear Dr. Sood,

Thank you for submitting your manuscript to PLOS One. After careful consideration, we feel that it has merit but does not fully meet PLOS One’s publication criteria as it currently stands. Therefore, we invite you to submit a revised version of the manuscript that addresses the points raised during the review process.

Please address all commenst from reviwer 1 - you can find these attached as a separate document.

Please submit your revised manuscript by Nov 03 2026 11:59PM. If you will need more time than this to complete your revisions, please reply to this message or contact the journal office at plosone@plos.org. When you're ready to submit your revision, log on to https://www.editorialmanager.com/pone/ and select the 'Submissions Needing Revision' folder to locate your manuscript file.

- A letter that responds to each point raised by the academic editor and reviewer(s). You should upload this letter as a separate file labeled 'Response to Reviewers'.
- A marked-up copy of your manuscript that highlights changes made to the original version. You should upload this as a separate file labeled 'Revised Manuscript with Track Changes'.
- An unmarked version of your revised paper without tracked changes. You should upload this as a separate file labeled 'Manuscript'.

If you would like to make changes to your financial disclosure, please include your updated statement in your cover letter. Guidelines for resubmitting your figure files are available below the reviewer comments at the end of this letter.

If applicable, we recommend that you deposit your laboratory protocols in protocols.io to enhance the reproducibility of your results. Protocols.io assigns your protocol its own identifier (DOI) so that it can be cited independently in the future. For instructions see: https://journals.plos.org/plosone/s/submission-guidelines#loc-laboratory-protocols. Additionally, PLOS One offers an option for publishing peer-reviewed Lab Protocol articles, which describe protocols hosted on protocols.io. Read more information on sharing protocols at https://plos.org/protocols?utm_medium=editorial-email&utm_source=authorletters&utm_campaign=protocols.

As the corresponding author, your ORCID iD is verified in the submission system and will appear in the published article. PLOS supports the use of ORCID, and we encourage all coauthors to register for an ORCID iD and use it as well. Please encourage your coauthors to verify their ORCID iD within the submission system before final acceptance, as unverified ORCID iDs will not appear in the published article. *Only*  the individual author can complete the verification step; PLOS staff *cannot*  verify ORCID iDs on behalf of authors.

We look forward to receiving your revised manuscript.

Kind regards,

Benedikt Ley, PhD

Academic Editor

PLOS One

**Journal Requirements:**

If the reviewer comments include a recommendation to cite specific previously published works, please review and evaluate these publications to determine whether they are relevant and should be cited. There is no requirement to cite these works unless the editor has indicated otherwise.

[Note: HTML markup is below. Please do not edit.]

Reviewers' comments:

Reviewer's Responses to Questions

**Comments to the Author**

Reviewer #1: (No Response)

Reviewer #2: All comments have been addressed

**********

2. Is the manuscript technically sound, and do the data support the conclusions?

Reviewer #1: Yes

Reviewer #2: Yes

**********

3. Has the statistical analysis been performed appropriately and rigorously?

Reviewer #1: Yes

Reviewer #2: Yes

**********

4. Have the authors made all data underlying the findings in their manuscript fully available?

The PLOS Data policy

Reviewer #1: Yes

Reviewer #2: Yes

**********

5. Is the manuscript presented in an intelligible fashion and written in standard English?

Reviewer #1: Yes

Reviewer #2: Yes

**********

Reviewer #1: The authors have not addressed my comments, which in the original review were attached as a Word Document. I have reattached the same Word Document to this recommendation.

Reviewer #2: (No Response)

**********

what does this mean?). If published, this will include your full peer review and any attached files.

If you choose “no”, your identity will remain anonymous but your review may still be made public.

**Do you want your identity to be public for this peer review?**  For information about this choice, including consent withdrawal, please see our Privacy Policy

Reviewer #1: No

Reviewer #2: No

**********

[NOTE: If reviewer comments were submitted as an attachment file, they will be attached to this email and accessible via the submission site. Please log into your account, locate the manuscript record, and check for the action link "View Attachments". If this link does not appear, there are no attachment files.]

To ensure your figures meet our technical requirements, please review our figure guidelines: https://journals.plos.org/plosone/s/figures

You may also use PLOS’s free figure tool, NAAS, to help you prepare publication quality figures: https://journals.plos.org/plosone/s/figures#loc-tools-for-figure-preparation.

NAAS will assess whether your figures meet our technical requirements by comparing each figure against our figure specifications.

Attachment Submitted filename: Review PONE_D_26-25852 - Copy.docx

# Author comment: Author response to Decision Letter 2

**DOI:** 10.1371/journal.pone.0353640.r004

7 Oct 2026

Review of the manuscript: ‘Vivaray Hb pro noninvasive hemoglobin device: a prospective diagnostic accuracy study measuring agreement with a calibrated blood cell counter’ (PONE-D-26-25852)

This article assesses the agreement between a noninvasive hemoglobin measurement device and the gold standard laboratory CBC in measuring hemoglobin levels. The study was methodologically sound and provided an excellent report of the findings, complemented by a comprehensive introduction that gives readers sufficient background to understand the study's context. However, the discussion section (outside the Conclusion and Strengths and Weaknesses subheadings) seems to stray from the study's findings and needs a rework.

• The discussion should mainly focus on the findings of this study and put them into context around the broader non-invasive Hb monitoring landscape.

Answer: Yes, we agree that this would improve the Discussion. We have made changes.

• Some of the questions to consider for discussion: Is it “better” than the other non-invasive assays? Is this systematic bias also observed in other assays? Will these findings be reported to the manufacturer? Is this device fit for use? What use is it fit for? What, if any, is the clinical relevance of the observed systematic bias?

Answer: Thank you for the observations. We have modified the text to align with these suggestions. Specifically, we have answered the question

a. about the quality of the device compared to others (Paragraph beginning with the words "The performance of this device is similar to that of other noninvasive instruments:,

b. about the fitness of this device for use (Paragraph beginning with the words "Given that the bias is low at the clinically relevant..."), and

c. clinical relevance of the systematic bias (Paragraph beginning with the words "The bias varies at different hemoglobin levels...")

• The only discussion about the findings was in the last paragraph.

Answer: True! Apologies for the limited connection between findings and the discussion. Edits have been made.

• I would suggest integrating the first paragraph of the Conclusions as the beginning of the Discussion section, and adding discussions about relevant literature to each point mentioned in this paragraph.

Answer: Agreed. We have moved the paragraph to a location early in the discussion. The relocated paragraph still begins with the words "This large, prospective…"

• The discussion section should guide the readers to understand why, even with the systematic bias, the assessed device was still concluded to be suitable for certain kinds of utility by the authors. As it currently stands, the authors leave readers to draw their own conclusions by providing separate descriptions of the findings and the realities of other non-invasive Hb devices on the market.

Answer: Agreed. We think that with the edits the Discussion should have become better.

• Abstract

o The comment or interpretation section of the abstract should at least explain what the findings mean, in the context of Hb measurement.

Answer: The comments section of the abstract does state the implication of the device. We have made a slight edit, to make the conclusion a little clearer. The paragraph immediately prior to the comments section provides several relevant values, as also the statement about the bias in the clinically predominant range. This should allow readers to decide whether they are comfortable with the precision of the instrument.

• Introduction

o Second to last paragraph “The Vivaray Hb Pro device is a photoplethysmography instrument.”: Kindly add the manufacturer name and country in a bracket after the device’s name.

Answer: Reviewer 2 made the same comment. We have added manufacturer information. The text in the Introduction now adds the following sentence: It is manufactured by Bosch Global Software Technologies Pvt Ltd, Bengaluru (India).

• Methods

o Having a photograph or a diagram of the device would be most helpful for the readers

Answer: The photograph has been inserted. As corresponding and senior author I confess I am ambivalent about this suggestion. I was wondering if insertion of the picture might add an advertising flavor to the paper, but after consideration the picture has been put in.

o Figure 1: consider including details of exclusion reasons (age 8 years and below, etc.) to the figure

Answer: The figure (now Figure 2) has been modified accordingly

o “At the same time blood was drawn and sent to the pathology laboratory …”: venous blood? How much blood was taken?

Answer: The Methods section now says "At the same time 2 to 3 ml of blood was drawn and sent to the pathology laboratory" instead of "At the same time blood was drawn and sent to the pathology laboratory". This line is written in the paragraph immediately prior to the Statistics section.

o “… calibrated cell counter”: please include the details of the instruments used in this study. Coulter was referenced in the sections below this one, but it should be clearly stated.

Answer: Reviewer 2 had made the same observation. We have added information about the instruments.

• References

o Reference 1: Can you be so kind as to cite the report published by the WHO (reference 2) instead of the web page?

Answer: One citation (WHO) was copied directly from the paper, from the paragraph titled "Suggested citation".

The other WHO paper has been edited.

The two WHO references now read as follows:

1. WHO Global Anaemia estimates, 2025 Edition [Internet]. World Health Organization; 2025.; 2025 [cited 2026 Feb 20]. Available from: https://www.who.int/data/gho/data/themes/topics/anaemia_in_women_and_children

2. WHO global anaemia estimates: key findings, 2025. [Internet]. Geneva: World Health Organization; 2025.; 2025. Available from: https://iris.who.int/server/api/core/bitstreams/ffbc07c6-420c-4711-8589-2a7b6f3a3da2/content

Attachment Submitted filename: Response to reviewers v 5 Oct 2026.docx

# Peer review: Decision Letter 2

**DOI:** 10.1371/journal.pone.0353640.r005

**Authors:** Benedikt Ley

13 Oct 2026

Vivaray Hb pro noninvasive hemoglobin device: a prospective diagnostic accuracy study measuring agreement with a calibrated blood cell counter

PONE-D-26-25852R2

Dear Dr. Sood,

We’re pleased to inform you that your manuscript has been judged scientifically suitable for publication and will be formally accepted for publication once it meets all outstanding technical requirements.

Within one week, you’ll receive an e-mail detailing the required amendments. When these have been addressed, you’ll receive a formal acceptance letter and your manuscript will be scheduled for publication.

An invoice will be generated when your article is formally accepted. Please note, if your institution has a publishing partnership with PLOS and your article meets the relevant criteria, all or part of your publication costs will be covered. Please make sure your user information is up-to-date by logging into Editorial Manager at Editorial Manager® and clicking the ‘Update My Information' link at the top of the page. For questions related to billing, please contact billing support.

If your institution or institutions have a press office, please notify them about your upcoming paper to help maximize its impact. If they’ll be preparing press materials, please inform our press team as soon as possible -- no later than 48 hours after receiving the formal acceptance. Your manuscript will remain under strict press embargo until 2 pm Eastern Time on the date of publication. For more information, please contact onepress@plos.org.

Kind regards,

Benedikt Ley, PhD

Academic Editor

PLOS One

Additional Editor Comments (optional):

Reviewers' comments:

Reviewer's Responses to Questions

**Comments to the Author**

Reviewer #1: All comments have been addressed

Reviewer #2: All comments have been addressed

**********

2. Is the manuscript technically sound, and do the data support the conclusions?

Reviewer #1: Yes

Reviewer #2: Yes

**********

3. Has the statistical analysis been performed appropriately and rigorously?

Reviewer #1: Yes

Reviewer #2: Yes

**********

4. Have the authors made all data underlying the findings in their manuscript fully available?

The PLOS Data policy

Reviewer #1: Yes

Reviewer #2: Yes

**********

5. Is the manuscript presented in an intelligible fashion and written in standard English?

Reviewer #1: Yes

Reviewer #2: Yes

**********

Reviewer #1: (No Response)

Reviewer #2: (No Response)

**********

what does this mean?). If published, this will include your full peer review and any attached files.

If you choose “no”, your identity will remain anonymous but your review may still be made public.

**Do you want your identity to be public for this peer review?**  For information about this choice, including consent withdrawal, please see our Privacy Policy

Reviewer #1: No

Reviewer #2: No

**********

# Editor report: Acceptance letter

**DOI:** 10.1371/journal.pone.0353640.r006

**Authors:** Benedikt Ley

PONE-D-26-25852R2

PLOS One

Dear Dr. Sood,

I'm pleased to inform you that your manuscript has been deemed suitable for publication in PLOS One. Congratulations! Your manuscript is now being handed over to our production team.

At this stage, our production department will prepare your paper for publication. This includes ensuring the following:

* All references, tables, and figures are properly cited

* All relevant supporting information is included in the manuscript submission,

* There are no issues that prevent the paper from being properly typeset

You will receive further instructions from the production team, including instructions on how to review your proof when it is ready. Please keep in mind that we are working through a large volume of accepted articles, so please give us a few days to review your paper and let you know the next and final steps.

Lastly, if your institution or institutions have a press office, please let them know about your upcoming paper now to help maximize its impact. If they'll be preparing press materials, please inform our press team within the next 48 hours. Your manuscript will remain under strict press embargo until 2 pm Eastern Time on the date of publication. For more information, please contact onepress@plos.org.

You will receive an invoice from PLOS for your publication fee after your manuscript has reached the completed accept phase. If you receive an email requesting payment before acceptance or for any other service, this may be a phishing scheme. Learn how to identify phishing emails and protect your accounts at https://explore.plos.org/phishing.

If we can help with anything else, please email us at customercare@plos.org.

Thank you for submitting your work to PLOS One and supporting open access.

Kind regards,

PLOS One Editorial Office Staff

on behalf of

Dr. Benedikt Ley

Academic Editor

PLOS One
